# Identification of structural features of surface modifiers in engineered nanostructured metal oxides regarding cell uptake through ML-based classification

**DOI:** 10.3762/bjnano.15.75

**Published:** 2024-07-22

**Authors:** Indrasis Dasgupta, Totan Das, Biplab Das, Shovanlal Gayen

**Affiliations:** 1 Laboratory of Drug Design and Discovery, Department of Pharmaceutical Technology, Jadavpur University, Kolkata 700032, India

**Keywords:** Bayesian classification, cellular uptake, machine learning, nanoparticles (NPs)

## Abstract

Nanoparticles (NPs) are considered as versatile tools in various fields including medicine, electronics, and environmental science. Understanding the structural aspects of surface modifiers in nanoparticles that govern their cellular uptake is crucial for optimizing their efficacy and minimizing potential cytotoxicity. The cellular uptake is influenced by multiple factors, namely, size, shape, and surface charge of NPs, as well as their surface functionalization. In the current study, classification-based ML models (i.e., Bayesian classification, random forest, support vector classifier, and linear discriminant analysis) have been developed to identify the features/fingerprints that significantly contribute to the cellular uptake of ENMOs in multiple cell types, including pancreatic cancer cells (PaCa2), human endothelial cells (HUVEC), and human macrophage cells (U937). The best models have been identified for each cell type and analyzed to detect the structural fingerprints/features governing the cellular uptake of ENMOs. The study will direct scientists in the design of ENMOs of higher cellular uptake efficiency for better therapeutic response.

## Introduction

In recent years, the rapid advancement of nanotechnology has led to the widespread utilization of engineered nanostructured metal oxides (ENMOs) in various industrial and biomedical applications [1]. Nanoparticles (NPs) are described by the International Organization for Standardization as structures characterized by one, two, or three dimensions within the range of 1 to 100 nm [2]. The diminutive size of nanoparticles contributes to a significantly high surface area with respect to volume, resulting in enhanced reactivity, improved stability, and augmented functionality. In the field of nanomaterials, ENMOs are a notable subset. These nanoparticles consist of metal elements bonded with oxygen in intricate structures [3–4]. They exhibit exceptional physicochemical properties, which have led to their widespread utilization across various industries [5–6]. These nanomaterials are employed in, for example, electronics, cosmetics, and medicine because of their enhanced reactivity, large surface area, and tunable properties [7–8].

ENMOs can enter the human body [9] and engage with various biomacromolecules, including sugars, lipids, proteins, and nucleic acids. These biomolecules rapidly envelop the nanoparticle surface, creating a dynamic “protein corona”, which dictates the biological characteristics of the nanoparticles [10–11]. The composition of this corona is variable and relies on the concentrations and affinities of its different components to the nanoparticle surface. Cellular uptake of NPs happens through receptor-mediated active or passive transport across the cell membrane [12]. Excessive absorption by normal cells enables metal oxide nanoparticles to engage with various subcellular organelles, initiating diverse signaling pathways to generate a stress response within cells. This results in the production of free radicals. Ultimately, this cascade leads to damage to cellular organelles and the demise of the cell [13–15]. ENMOs have also been explored for potential diagnostic applications, particularly in targeting cancer cells [16–17]. To create target-specific NPs, researchers synthesized magnetofluorescent NPs with an iron oxide nanocore decorated with organic compounds and investigated their cellular uptake across various human cell types [18]. However, determining the cellular absorption of functionalized nanoparticles in different human cell types is a laborious, expensive, and time-consuming task. Computational analysis of experimentally obtained cellular uptake data for ENMOs provides a systematic approach to gain insights for modifying them for specific purposes. In recent times, these computational methods have gained popularity as they are more cost-effective and independent alternatives to experimental procedures [19–21].

**Figure 1:**
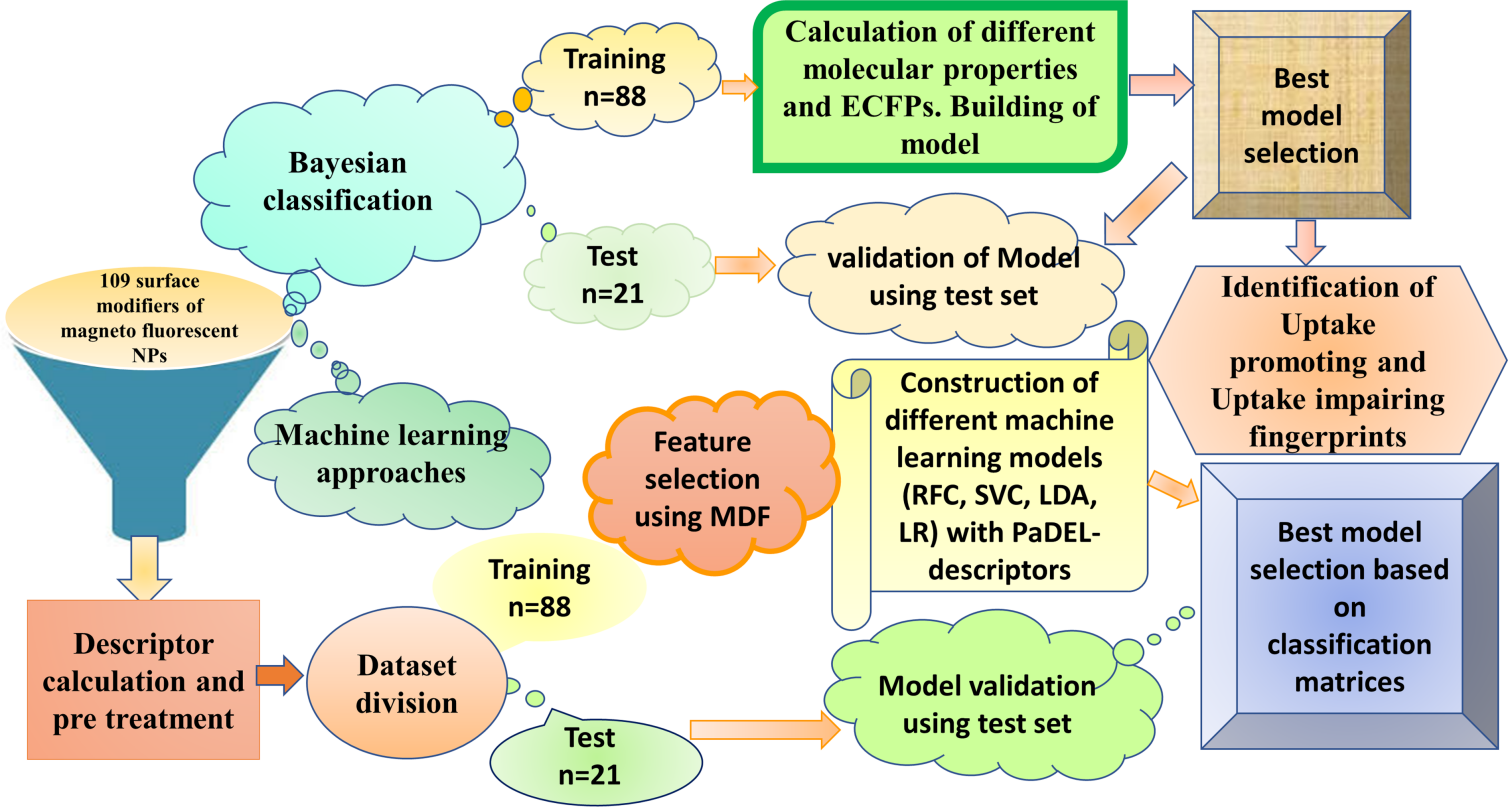
Workflow of the current study for cellular uptake of ENMOs involving different approaches such as Bayesian classification and machine learning. The similar training (*n* = 88) and test set (*n* = 21) were used in different analyses.

Understanding the structural features related to the surface modifiers of ENMOs that influence their uptake in human cell lines is crucial for designing nanomaterials with enhanced bioavailability. The surface modifiers are, in general, chemical groups or molecules that are attached to the surface of ENMOs to modify their properties and, specifically, the cellular uptake. A lot of computational studies (Table 1) have been reported using nanoscale quantitative structure–activity relationship (nano-QSAR) models (predominantly regression-based) that specifically employ the cellular uptake in the PaCa2 cell line [22–27]. In the current study, we have performed a distinctive approach by developing nano-QSAR machine learning-based classification models that encompass not only the cellular uptake data of the PaCa2 cell line but also the two additional cell lines HUVEC and U937. The primary objective is to find the structural fingerprints/features that govern cellular uptake selectivity for each cell line. The selective surface modifications of ENMOs could enhance the affinity of the nanoparticles for certain cell types while reducing the uptake by non-target cells. This is particularly important for in vivo applications where non-specific uptake by the reticuloendothelial system (e.g., liver and spleen) can reduce the efficacy of the nanoparticles. The workflow of the current study is shown in Figure 1. The insights gained from this study hold significant implications for the rational design of ENMOs with tailored properties for biomedical applications, ensuring their higher efficiency.

**Table 1:** Comparison of statistical parameters of the present model with previous studies for the cellular uptake of ENMOs.

S. no.	Cell line	*n*_train_	*n*_test_	Model^a^	Statistical parameters^b^	Ref

Regression-based QSAR						

1	PaCa2	87	22	—	*R*^2^_Te_ = 0.72; RMSE_Te_ = 0.18	[22]
2	PaCa2	90	19	MLR	*R*^2^_Tr_ = 0.934; RMSE_Tr_ = 0.121; *R*^2^_Te_ = 0.943; RMSE_Te_ = 0.214	[23]
3	HUVEC	87	21	BRANNLP & MLREM	*R*^2^_Tr_ = 0.55; RMSE_Tr_ = 0.38; *R*^2^_Te_ = 0.72; RMSE_Te_ = 0.30	[24]
	PaCa2				*R*^2^_Tr_ = 0.64; RMSE_Tr_ = 0.26; *R*^2^_Te_ = 0.62; RMSE_Te_ = 0.32	
4	PaCa2	91	18	Monte Carlo regression	*R*^2^_Tr_ = 0.76; RMSE_Tr_ =0.19; *R*^2^_Te_ = 0.86; RMSE_Te_ = 0.14	[25]
5	PaCa2	87	22	MLR	*R*^2^_Tr_ = 0.945; RMSE_Tr_ = 0.13; *R*^2^_Te_ = 0.897; RMSE_Te_ = 0.18	[26]
6	PaCa2	89	20	PLS	LV = 5; *R*^2^_Tr_ = 0:806; *Q*^2^_LOO_ = 0.758; RMSE_Tr_ = 0.20; *Q*^2^_F1_ = *R*^2^_Te_ = 0.879; *Q*^2^_F2_ = 0.868; RMSE_Te_ = 0.12	[27]
7	HUVEC	87	21	MLR Bayesian regularized neural network	*R*^2^_Tr_ = 0.74; RMSE_Tr_ = 0.34; *R*^2^_Te_ = 0.63; RMSE_Te_ = 0.36 (linear model) R^2^_Tr_ = 0.70; RMSE_Tr_ = 0.30; *R*^2^_Te_ = 0.66; RMSE_Te_ = 0.33 (nonlinear model)	[28]
	PaCa2				*R*^2^_Tr_ = 0.76; RMSE_Tr_ = 0.19; *R*^2^_Te_ = 0.79; RMSE_Te_ = 0.24 (linear model) *R*^2^_Tr_ = 0.77; RMSE_Tr_ = 0.15; *R*^2^_Te_ = 0.54; RMSE_Te_ = 0.28 (nonlinear model)	
8	PaCa2	83	21	MLR	*R*^2^_Tr_ = 0.974; RMSE_Tr_ = 0.067; *R*^2^_Te_ = 0.944; RMSE_Te_ = 0.109	[29]
	HUVEC				*R*^2^_Tr_ = 0.973; RMSE_Tr_ = 0.100; *R*^2^_Te_ = 0.966; RMSE_Te_ = 0.104	
	U937				*R*^2^_Tr_ = 0.977; RMSE_Tr_ = 0.019; *R*^2^_Te_ = 0.938; RMSE_Te_ = 0.023	
9	PaCa2	36 27 15	9 7 3	MLR	*R*^2^_Tr_ = 0.792; *Q*^2^_LOO_ = 0.765; RMSE_Tr_ = 1929.40 *R*^2^_Te_ = 0.954; *Q*^2^_ext_ = 0.908; RMSE_Te_ = 581.646 (Model 1) *R*^2^_Tr_ = 0.857; *Q*^2^_LOO_ = 0.735; RMSE_Tr_ = 1649.077 *R*^2^_Te_ = 0.961; *Q*^2^_ext_ = 0.923; RMSE_Te_ = 1083.365 (Model 2) *R*^2^_Tr_ = 0.819; *Q*^2^_LOO_ = 0.739; RMSE_Tr_ = 1683.908 *R*^2^_Te_ = 0.863; *Q*^2^_ext_ = 0.821; RMSE_Te_ = 1683.908 (Model 3)	[30]
10	PaCa2	87	22	PLS	LV = 4; *R*^2^_Tr_ = 0.814; *Q*^2^_LOO_ = 0.782; RMSE_Tr_ = 0.198; *Q*^2^_F1_ = 0.893; *Q*^2^_F2_ = 0.749	[31]
	HUVEC				LV = 5; *R*^2^_Tr_ = 0.782; *Q*^2^_LOO_ = 0.733; RMSE_Tr_ = 0.299; *Q*^2^_F1_ = 0.704; *Q*^2^_F2_ = 0.668	
	U937				LV = 5; *R*^2^_Tr_ = 0.667; *Q*^2^_LOO_ = 0.539; RMSE_Tr_ = 0.077; *Q*^2^_F1_ = 0.602; *Q*^2^_F2_ = 0.506	
11	HUVEC	87	22	MLR	*R*^2^_Tr_ = 0.852; RMSE_Tr_ = 0.235; *R*^2^_Te_ = 0.822; RMSE_Te_ = 0.241	[32]
	PaCa2				*R*^2^_Tr_ = 0.905; RMSE_Tr_ = 0.130; *R*^2^_Te_ = 0.885; RMSE_Te_ = 0.140	

Classification-based QSAR						

12	PaCa2	—	—	DTB	Se_Tr_ = 1.000; Sp_Tr_ = 0.974; ACC_Tr_ = 0.988; MCC_Tr_ = 0.980 Se_Te_ = 0.882; Sp_Te_ = 1.000; ACC_Te_ = 0.926; MCC_Te_ = 0.860	[26]
13	PaCa2	—	—	DTF	Se_Tr_ = 1.000; Sp_Tr_ = 1.000; ACC_Tr_ = 1.000; MCC_Tr_ = 1.000 Se_Te_ = 0.875; Sp_Te_ = 0.909; ACC_Te_ = 0.889; MCC_Te_ = 0.780	
14	PaCa2	89	20	RF	Se_Tr_ = 0.958; Sp_Tr_ = 0.976; ACC_Tr_ = 0.966; MCC_Tr_ = 0.933 Se_Te_ = 0.909; Sp_Te_ = 1.000; ACC_Te_ = 0.950; MCC_Te_ = 0.905	[33]
15	PaCa2	88	21	Bayesian classification	Se_Tr_ = 0.980; Sp_Tr_ = 0.865; Conc._Tr_ = 0.932; ROC_Tr_ = 0.765; Se_Te_ = 1.000; Sp_Te_ = 0.800; Conc._Te_ = 0.905; ROC_Te_ = 0.891	our model
	HUVEC			SVC	Se_Tr_ = 0.952; ACC_Tr_ = 0.875; MCC_Tr_ = 0.761; ROC_Tr_ = 0.969; Se_Te_ = 0.833; ACC_Te_ = 0.857; MCC_Te_ = 0.716; ROC_Te_ = 0.870	
	U937			LDA	Se_Tr_ = 0.827; ACC_Tr_ = 0.716; MCC_Tr_ = 0.400; ROC_Tr_ = 0.735; Se_Te_ = 0.833; ACC_Te_ = 0.667; MCC_Te_ = 0.304; ROC_Te_ = 0.630	

^a^Various models reported as follows: MLR = multiple linear regression; RMSEP = root mean square error of prediction; Conc. = concordance, RF = random forest; SVC = support vector classifier, LDA = linear discriminant analysis; DTB = decision tree boost; DTF = decision tree forest; PLS = partial least squares; BRANNLP = Bayesian regularization artificial neural network, using Gaussian priors, MLREM = multiple linear regression with expectation maximization; ^b^different statistical parameters reported as follows: *R*^2^ = correlation coefficient, ACC = accuracy, MCC = Matthews correlation coefficient; ROC = receiver operating characteristic; RMSE = root mean square error; *Q*^2^_LOO_ = cross-validated correlation coefficient; LV = latent variables; Se = sensitivity; Sp = specificity.

## Materials and Methods

### Preparation of datasets

The current study was performed employing the experimental cellular uptake data of 109 chemically attached surface modifiers of ENMOs (monocrystalline magnetic nanoparticles having overall size of 38 nm and an average of 60 ligands per nanoparticle, indicating a consistent level of attachment across different preparations) regarding human pancreatic ductal adenocarcinoma cells (PaCa2), human umbilical vein endothelial cells (HUVEC), and the human monocyte lymphoma cell line U937 [34]. PaCa2 cells are derived from a human pancreatic tumor and are adherent and epithelial in nature, providing insights into the uptake and behavior of nanoparticles in pancreatic cancer. HUVEC cells are endothelial cells derived from the vein of the umbilical cord to study vascular biology and endothelial function. U937 is a human cell line used as a model for monocyte/macrophage differentiation. The cellular uptake was represented by log_10_[NP]/cell, in which the concentration was represented in picomoles per cell. In order to classify the higher-uptake (assigned as “1”) and lower-uptake (assigned as “0”) surface modifiers of ENMOs, the average values of log_10_[NP]/cell were considered as cut-off value (Supporting Information File 1, Table S1). Thus, 62 higher-uptake and 47 lower-uptake (in the case of PaCa2 cell line); 54 higher-uptake and 55 lower-uptake (in the case of HUVEC cell line), and 64 higher-uptake and 45 lower-uptake (in the case of U937 cell line) surface modifiers of ENMOs were included in the modelling. The whole dataset was divided based on the “Diverse molecule” method in Discovery studio 3.0 software [35] into 88 modifiers in the training set (70%) and 21 modifiers in the test set (30%) for the different classification-based QSAR analyses.

### Bayesian classification study

Bayesian classification was carried out via the “Create Bayesian model” protocol in Discovery Studio 3.0 [35]. To develop a model, various descriptors were collected, including molecular weight (MW), *n*-octanol/water partition coefficient (ALogP), number of aromatic rings (nAR), number of rings (nR), number of rotatable bonds (nBonds), number of hydrogen bond donors (nHBDs), and the number of hydrogen bond acceptors (nHBAs) [36]. Extended-connectivity fingerprints (ECFPs) or functional-class fingerprints (FCFPs) were also used for the Bayesian analysis. ECFPs are circular fingerprints that capture precise substructural features of molecules, making them suitable for predicting molecular activity and similarity search [37]. They are generated through an iterative process based on the Morgan algorithm, which assigns numeric identifiers to each atom in a molecule and updates these identifiers through several iterations. In contrast, FCFPs focus on capturing functional class information, reflecting the pharmacophore roles of atoms. Both ECFPs and FCFPs are highly customizable and have been widely adopted for various scientific applications [38–39]. The molecules from the training set were used for constructing the model, and the molecules from the test set were used for the validation. The resulting model’s statistical properties were assessed using the fivefold cross-validation procedure. Additionally, the model’s quality was evaluated by looking at the receiver operating characteristic (ROC) plot as well as specificity, sensitivity, and accuracy values [40–42].

### Development of other machine learning models

#### Calculation of descriptors and data pre-treatment

The training set of 88 and the test set of 21 surface modifiers from Bayesian classification analysis were used for the development of other machine learning models. Different classes of 2D descriptors were calculated using PaDEL-Descriptor [43]. The data pre-treatment tool (Data Pre-TreatmentGUI 1.2 from DTC laboratory, Jadavpur University, available at http://teqip.jdvu.ac.in/QSAR_Tools/) removed some descriptors (intercorrelation cutoff > 0.90, variance cutoff < 0.0001) [44].

#### Feature selection

Finding the minimum number of significant features or variables in the descriptor form is a vital step in the interpretation of a ML model [45]. In our current study, the most discriminating features selection method (MDF_Identifier-v1.0 accessible at https://sites.google.com/jadavpuruniversity.in/dtc-lab-software/home) was used to find out the minimum number of required features that are responsible for classifying higher-uptake and lower-uptake surface modifiers in the case of three cell lines [46]. The descriptors that had greater values of absolute difference were taken as significant features for a particular cell line. For the study of the PaCa2 cell line, we selected ten descriptors (Supporting Information File 1, Table S2) that had an absolute difference value greater than or equal to 0.31. Similarly, for the study of HUVEC and U937 cell lines, we selected, respectively, eight (Supporting Information File 1, Table S3) and eleven descriptors (Supporting Information File 1, Table S4) that had an absolute difference greater than or equal to 0.39 and 0.19, respectively. The specific values were determined through empirical analysis, ensuring that the selected descriptors provide the best predictive performance for each cell line.

### ML model development and analysis

Four classification-based ML models, namely, random forest classifier (RFC), support vector classifier (SVC), linear discriminant analysis (LDA), and logistic regression (LR) were developed in the current analysis. These models were developed using the optimized hyper parameters in the Scikit Learn package. The ML models were built by utilizing the ML classifier tool (https://sites.google.com/jadavpuruniversity.in/dtc-lab-software/home/machine-learning-model-development-guis) [47]. For applicability domain analysis, the leverages of the training and test set compounds were calculated. The applicability domain analysis was performed with the help of Hi_Calculator-v2.0, accessible at https://sites.google.com/jadavpuruniversity.in/dtc-lab-software/home [48].

## Results and Discussion

### Bayesian classification study for the three cell lines

#### PaCa2 cell line

**Figure 2:**
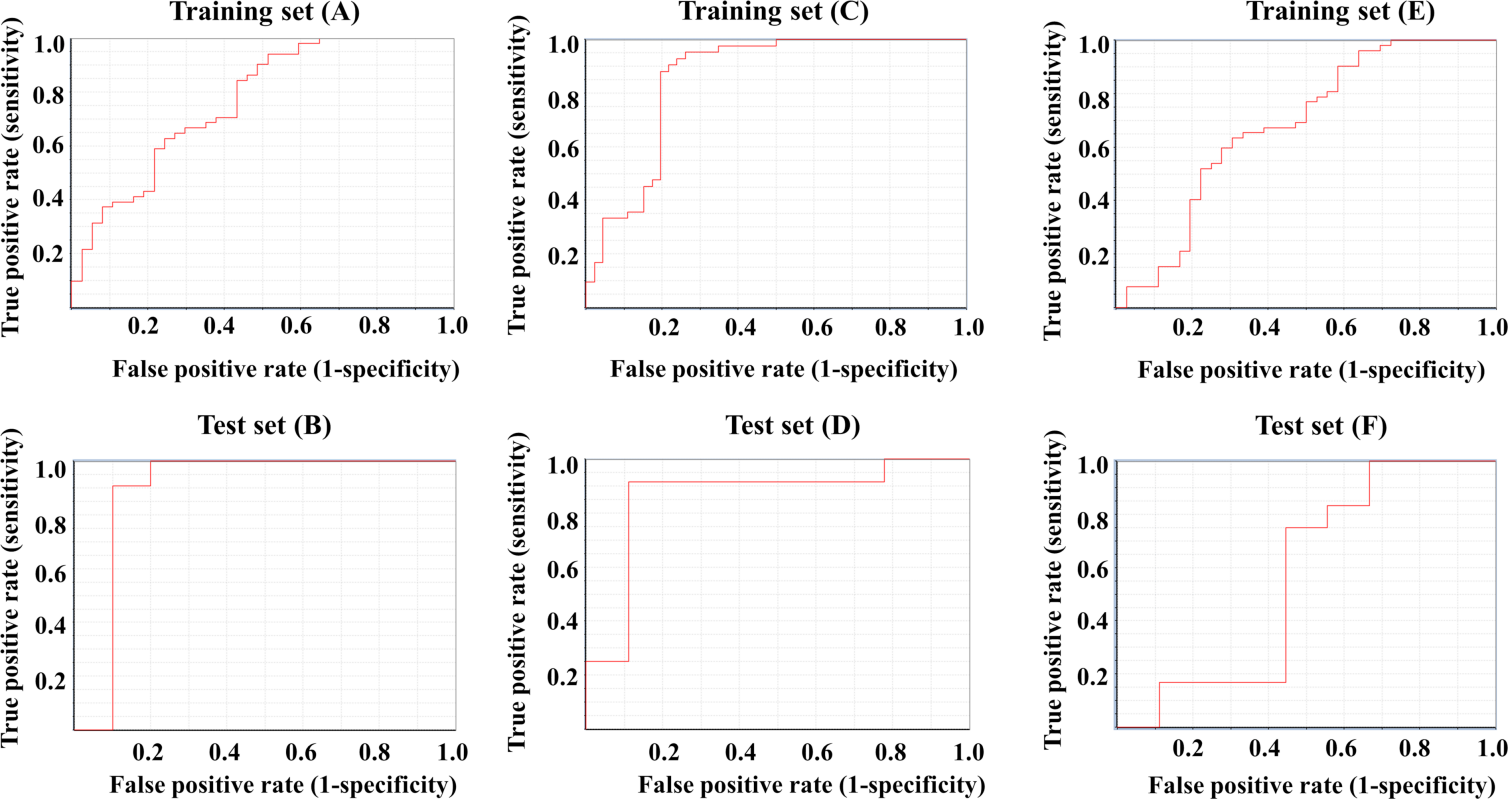
Receiver operating characteristic plots of the training set (A, C, E) and test set (B, D, F) for the Bayesian classification analysis in the case of PaCa2 cell line (A, B), HUVEC (C, D) and U937 (E, F) cell line.

Initially, a Bayesian classification study was carried out in order to build a classification-based QSAR model. The test set was developed with 21 molecules, whereas the training set was developed with 88 molecules. Figure 2A,B depict the ROC curves for the compounds in the training and test set of the surface modifiers of ENMOs in the PaCa2 cell line. Various statistical criteria, such as concordance, specificity, and sensitivity, were examined to characterize the model (Table 2). The developed Bayesian model has a fivefold cross-validated ROC of 0.765, indicating the model’s validity. The ROC for the test set is 0.891, indicating an acceptable external validation result. The training set’s statistical results are summarized in Table 2, showing a strong 98% sensitivity, 86.5% specificity, and 93.2% overall concordance.

**Table 2:** Validation parameters of the generated classification-based Bayesian model for different cell lines.

Cell line	Set	TP^a^	FN^b^	FP^c^	TN^d^	Sen^e^	Spec^f^	Conc^g^	ROC^h^

PaCa2	training	50	1	5	32	0.980	0.865	0.932	0.765
	test	11	0	2	8	1.000	0.800	0.905	0.891
HUVEC	training	39	3	5	41	0.929	0.891	0.909	0.854
	test	10	2	1	8	0.833	0.889	0.857	0.861
U937	training	52	0	14	22	1.000	0.611	0.841	0.682
	test	6	6	4	5	0.500	0.556	0.524	0.565

^a^True positive; ^b^false negative; ^c^false positive; ^d^true negative; ^e^sensitivity; ^f^specificity; ^g^concordance; ^h^receiver operating characteristic.

**Figure 3:**
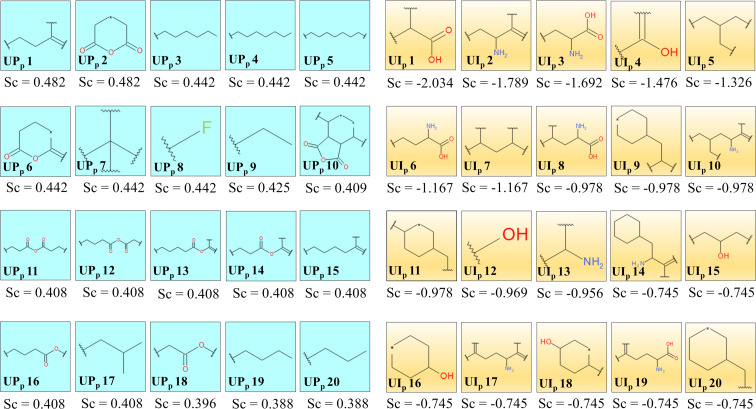
Uptake-promoting (UP_p_ 1–UP_p_ 20) and uptake-impairing (UI_p_ 1–UI_p_ 20) fingerprints from the Bayesian study (PaCa2 cell line). Sc denotes the Bayesian score of the corresponding fingerprints.

Twenty uptake-promoting (UP_p_ 1–UP_p_ 20) and twenty uptake-impairing (UI_p_ 1–UI_p_ 20) structural features/fingerprints were generated by the Bayesian model of 109 surface modifiers. As seen in Figure 3, uptake-promoting and uptake-impairing fingerprints can be matched into fewer structural features/fingerprint groups, as explained below.

A long aliphatic carbon chain of the surface modifiers in ENMOs is highly beneficial for improved uptake in the PaCa2 cell line as suggested by the fingerprints UP_p_ 3, UP_p_ 4, UP_p_ 5, UP_p_ 9, UP_p_ 19, and UP_p_ 20. For example, surface modifiers **68** and **73** have these essential fingerprints and exhibit higher uptake (Supporting Information File 1, Figure S1). The uptake of ENMOs with surface modifiers like **49** is also high because of the presence of long-chain aliphatic anhydride-like fingerprints such as in UP_p_ 11, UP_p_ 12, UP_p_ 13, UP_p_ 14, UP_p_ 16, and UP_p_ 18. The fingerprints UP_p_ 2 and UP_p_ 6 share the similarity of a dihydro-2*H*-pyran-2,6(3*H*)-dione structure. These fingerprints are seen in surface modifiers **18** and **28**.

The uptake-impairing fingerprints UI_p_ 12, UI_p_ 15, UI_p_ 16, and UI_p_ 18 indicate the presence of aliphatic/cyclic alcohol-like structures in the surface modifiers, and a negative impact on cell uptake of ENMOs is shown in the case of surface modifier **59**. Similarly, fingerprints UI_p_ 2, UI_p_ 3, UI_p_ 6, UI_p_ 8, UI_p_ 13, and UI_p_ 19 represent the presence of amino groups with a possible carboxyl functionality. Such fingerprints are observed in surface modifier **101**. The fingerprints UI_p_ 9, UI_p_ 11, UI_p_ 14, and UI_p_ 20, having a cyclohexane ring (e.g., **90**), also reduce the uptake of ENMOs in the PaCa2 cell line.

#### HUVEC cell line

**Figure 4:**
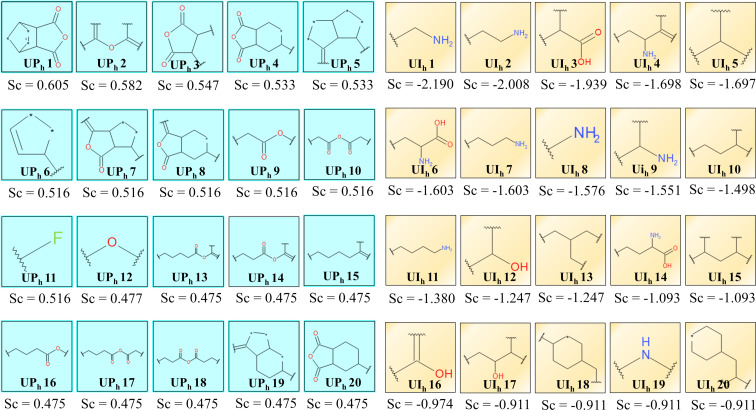
Uptake-promoting (UP_h_ 1–UP_h_ 20) and uptake-impairing (UI_h_ 1–UI_h_ 20) fingerprints from the Bayesian study (HUVEC cell line). Sc denotes the Bayesian score of the corresponding fingerprints.

In the case of the HUVEC cell line, the fivefold cross-validated ROC values for the training set and test set are 0.854 and 0.861, respectively. The ROC plots (Figure 2C,D) have been generated to justify the internal and external predictability of the model. The statistical factors sensitivity, specificity, and concordance are reported in Table 2. The presence of the aliphatic anhydride-like fingerprints UP_h_ 9, UP_h_ 10, UP_h_ 17, and UP_h_ 18 (Figure 4) in the surface modifiers promotes uptake in the HUVEC cell line (Supporting Information File 1, Figure S3). As discussed previously, similar fingerprints are also important for the uptake in the case of the PaCa2 cell line. Furthermore, fingerprints like UP_h_ 13, UP_h_ 14 and UP_h_ 16, having ester functionality, are also responsible for a higher uptake of ENMOs in the HUVEC cell line. Fingerprints having a dihydrofuran-2,5-dione scaffold (UP_h_ 3, UP_h_ 4, UP_h_ 8, and UP_h_ 20) in the surface modifiers are important for the higher uptake of ENMOs in the HUVEC cell line, too. This is shown in the case of surface modifier **30** (Figure S3, Supporting Information File 1). The presence of fingerprints like UP_h_ 1, UP_h_ 5, and UP_h_ 7 are also important for the uptake of ENMOs in the HUVEC cell line as shown in the case of surface modifier **46**.

However, fingerprints containing aliphatic amino functionality (UI_h_ 1, UI_h_ 2, UI_h_ 7, UI_h_ 8, UI_h_ 9, and UI_h_ 11) have a deleterious effect on the uptake of ENMOs in the HUVEC cell line, as demonstrated in in the case of surface modifier **74** (Supporting Information File 1, Figure S4). The fingerprints UI_h_ 5, UI_h_ 10, UI_h_ 13, UI_h_ 15, and UI_h_ 18 with a branched aliphatic structure have a negative impact on the uptake of ENMOs. As discussed previously in the case of the PaCa2 cell line, aliphatic alcohol-related fingerprints, such as UI_h_ 12 and UI_h_ 17, also impair uptake in the HUVEC cell line. Other fingerprints responsible for impairing uptake in the HUVEC cell line include UI_h_ 3, UI_h_ 6, and UI_h_ 14. These fingerprints suggest uptake impairment of ENMOs by the presence of a carboxyl group with or without amino functionality in the surface modifiers as shown in Figure S4 (Supporting Information File 1).

#### U937 cell line

The ROC curves for the U937 cell line are shown in Figure 2E,F for training and test set separately, and the statistical parameters for the model are shown in Table 2. The training set has sensitivity = 1.000, specificity = 0.611, and concordance = 0.841. The test set has sensitivity = 0.841, specificity = 0.556, and concordance = 0.524. The statistical quality of the Bayesian classification model for the U937 cell line is inferior compared to the models for the other cell lines. The training and test sets have also shown lower ROC scores of 0.682 and 0.565, respectively.

**Figure 5:**
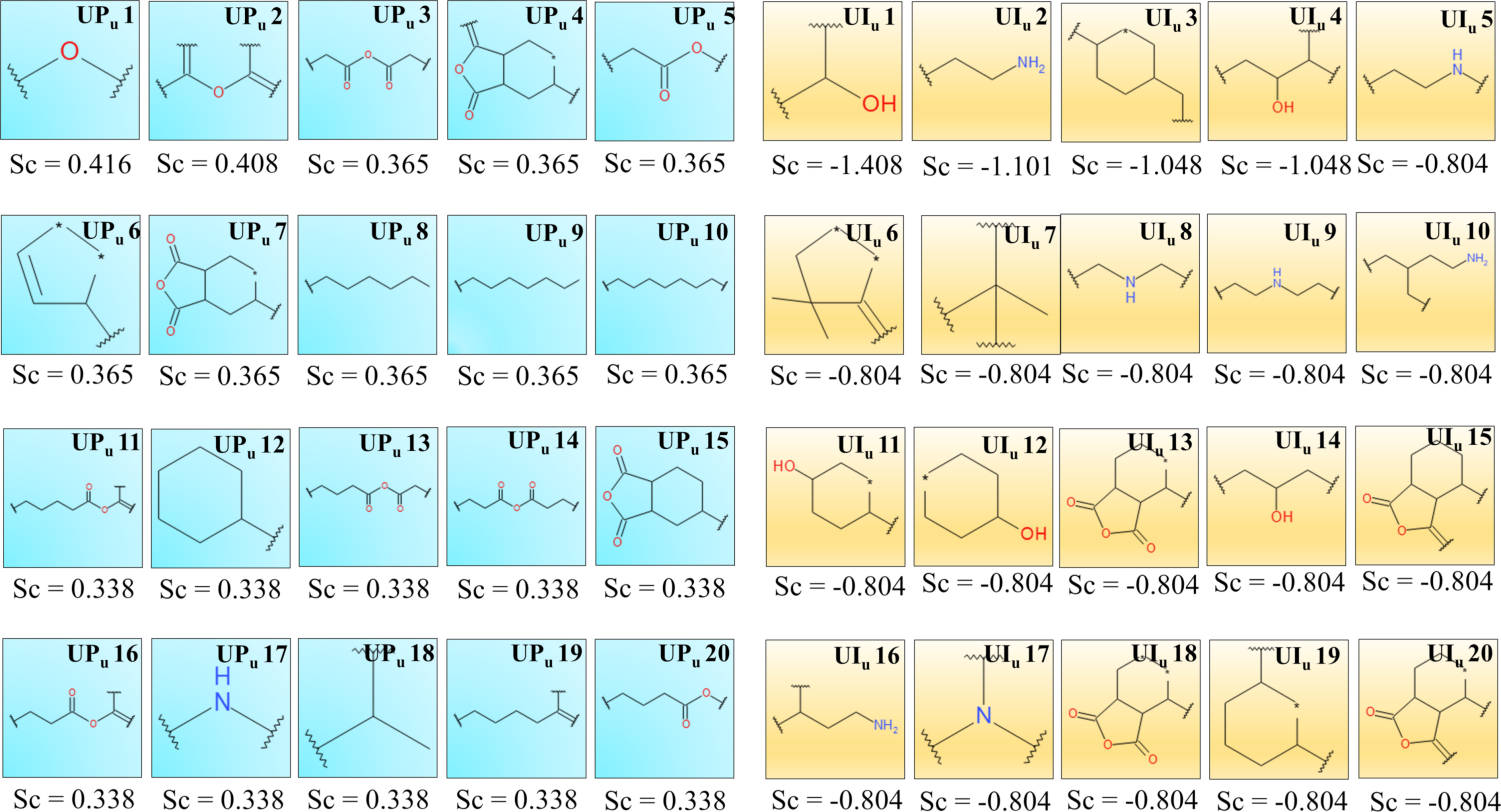
Uptake-promoting (UP_u_ 1–UP_u_ 20) and uptake-impairing (UI_u_ 1–UI_u_ 20) fingerprints from the Bayesian study (U937 cell line). Sc denotes the Bayesian score of the corresponding fingerprints.

For U937, the Bayesian model also yielded 20 favorable fingerprints (UP_u_ 1–UP_u_ 20) and 20 unfavorable fingerprints (UI_u_ 1–UI_u_ 20) using ECFP_6 fingerprint descriptors, as shown in Figure 5. The fragments UP_u_ 8–UP_u_ 10 highlight the significance of the long aliphatic chain for the increased uptake of ENMOs as shown in the case of surface modifier **68**. The fingerprints having anhydride functionality, for example, UP_u_ 3, UP_u_ 11, UP_u_ 13, and UP_u_ 14, are important for the uptake of ENMOs in the case of the U937 cell line (surface modifier **49** in Supporting Information File 1, Figure S5). The presence of dihydrofuran-2,5-dione scaffold-like structures in fingerprints including UP_u_ 4, UP_u_ 7, and UP_u_ 15 is also important for the uptake of ENMOs in the U937 cell line (surface modifier **54** in in Supporting Information File 1, Figure S5). A similar feature is found to be important also in the case of the HUVEC cell line as discussed previously. Other fingerprints promoting uptake in the U937 cell line (UP_u_ 5, UP_u_ 16, and UP_u_ 20) have an ester functionality (Supporting Information File 1, Figure S5). The higher uptake of ENMOs with surface modifier **86** is due to the presence of fingerprints UP_u_ 12 and UP_u_ 18.

The uptake-impairing fingerprints UI_u_ 1, UI_u_ 4, UI_u_ 11, UI_u_ 12, and UI_u_ 14 indicate the presence of aliphatic alcohol functionality. The presence of primary or secondary amino groups (UI_u_ 2, UI_u_ 5, UI_u_ 8, UI_u_ 9, UI_u_ 10, and UI_u_ 16) also has a negative impact on the uptake of ENMOs in the U937 cell line as illustrated in the case of surface modifier **22** (Supporting Information File 1, Figure S6).

### Other machine learning models

**Figure 6:**
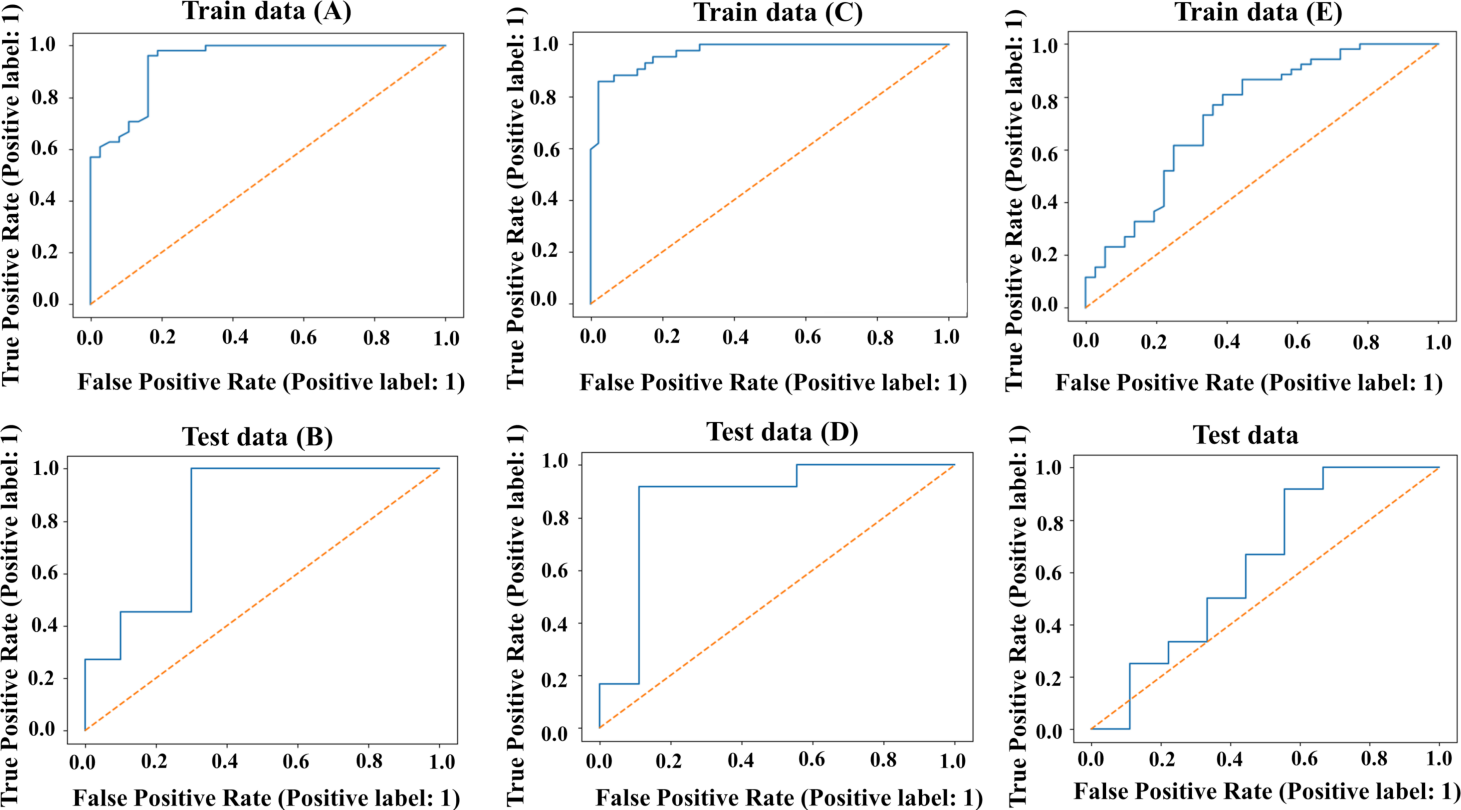
Receiver operating characteristic plots of training set (A, C, E) and test set (B, D, F) for the ML-based classification models in the case of PaCa2 Cell line (A, B), HUVEC (C, D) and U937 (E, F) Cell line.

Other classification-based machine learning (ML) models (RFC, SVC, LDA, and LR) were also developed individually for the three cell lines (PaCa2, HUVEC, and U937) for the 109 surface modifiers of magnetofluorescent ENMOs. Various statistical parameters were evaluated for the selection of the best ML model. Regarding classification-based validation measures (Table 3), the random forest (RF) model exhibited the highest performance for the PaCa2 cell line, while the support vector classifier (SVC) model demonstrated superior performance for the HUVEC cell line. The linear discriminant analysis (LDA) model performed best for the U937 cell line. Figure 6A–F depicts the ROC curves for the compounds in the training and test sets of each cell line. The best ML model (RF) for the PaCa2 cell line has fivefold cross-validated ROC values of 0.939 for the training set and 0.818 for the test set, which indicates an acceptable internal and external validation result. The best ML model (SVC) for the HUVEC cell line has fivefold cross-validated ROC values of 0.969 for the training set and 0.870 for the test set, indicating that the internal and external validation result is acceptable. Last, the best ML model for the U937 cell line (LDA) has fivefold cross-validated ROC values of 0.735 for the training set and 0.630 for the test set. The detailed statistical analysis is presented in Table 3. The applicability domain analysis was also performed in order to check the chemical space of training and test set of surface modifiers of ENMOs. Based on the leverage calculation, surface modifiers **16**, **48**, **78**, **79**, **83**, **86**, and **107** from the training set and **82** from the test set are outliers for the classification model of the cellular uptake data for PaCa2 cell line. Similarly, based on the leverage calculation, surface modifiers **48**, **83**, **86**, and **107** in the training set and **13**, **40**, and **109** in the test set are outliers for the classification model of the cellular uptake data for HUVEC cell line. For the developed classification model for the U937 cell line, surface modifiers **48**, **59**, **80**, **83**, and **97** from the training set and **10**, **82**, **95**, and **109** from the test set are outliers.

**Table 3:** Validation parameters of the classification-based ML models for PaCa2, HUVEC, and U937 cell line.

Cell line	Model Type	Set	Accuracy	Precision	Recall	F1 score	MCC^a^	Cohen’s k	AUC-ROC^b^

PaCa2	RFC	training	0.852	0.807	0.980	0.885	0.710	0.684	0.939
		test	0.857	0.786	1.000	0.880	0.742	0.710	0.818
	SVC	training	0.739	0.750	0.823	0.785	0.457	0.454	0.791
		test	0.619	0.636	0.636	0.636	0.236	0.236	0.536
	LDA	training	0.818	0.769	0.980	0.862	0.646	0.607	0.862
		test	0.857	0.786	1.000	0.880	0.742	0.710	0.855
	LR	training	0.830	0.781	0.980	0.870	0.667	0.632	0.874
		test	0.857	0.786	1.000	0.880	0.742	0.710	0.873
HUVEC	RFC	training	0.841	0.780	0.929	0.848	0.695	0.684	0.970
		test	0.905	0.917	0.917	0.917	0.806	0.806	0.889
	SVC	training	0.875	0.816	0.952	0.879	0.761	0.751	0.969
		test	0.857	0.909	0.833	0.870	0.716	0.712	0.870
	LDA	training	0.841	0.792	0.905	0.844	0.690	0.683	0.934
		test	0.905	0.917	0.917	0.917	0.806	0.806	0.889
	LR	training	0.830	0.765	0.929	0.839	0.676	0.662	0.891
		test	0.905	0.917	0.917	0.917	0.806	0.806	0.944
U937	RF	training	0.739	0.754	0.827	0.789	0.451	0.448	0.744
		test	0.619	0.667	0.667	0.667	0.222	0.222	0.611
	SVC	training	0.693	0.698	0.846	0.765	0.347	0.334	0.687
		test	0.667	0.667	0.833	0.741	0.304	0.290	0.630
	LDA	training	0.716	0.729	0.827	0.775	0.400	0.394	0.735
		test	0.667	0.667	0.833	0.741	0.304	0.290	0.630
	LR	training	0.693	0.698	0.846	0.765	0.347	0.334	0.699
		test	0.667	0.667	0.833	0.741	0.304	0.290	0.685

^a^Matthew’s correlation coefficient; ^b^area under the receiver operating characteristic curve.

#### Interpretation of the descriptors of the best ML based classification models

According to OECD Principle 5 on the validation of QSAR models, it is very important to give a mechanistic interpretation of the descriptors that have a significant contribution to the model output [49]. In the current study, SHapley Additive exPlanation (SHAP) analysis was performed on the training datasets for the three cell lines using the best identified models. An increased value with a greater spreading from the mean identify the most important descriptors in the SHAP summary plot.

#### PaCa2 cell line

**Figure 7:**
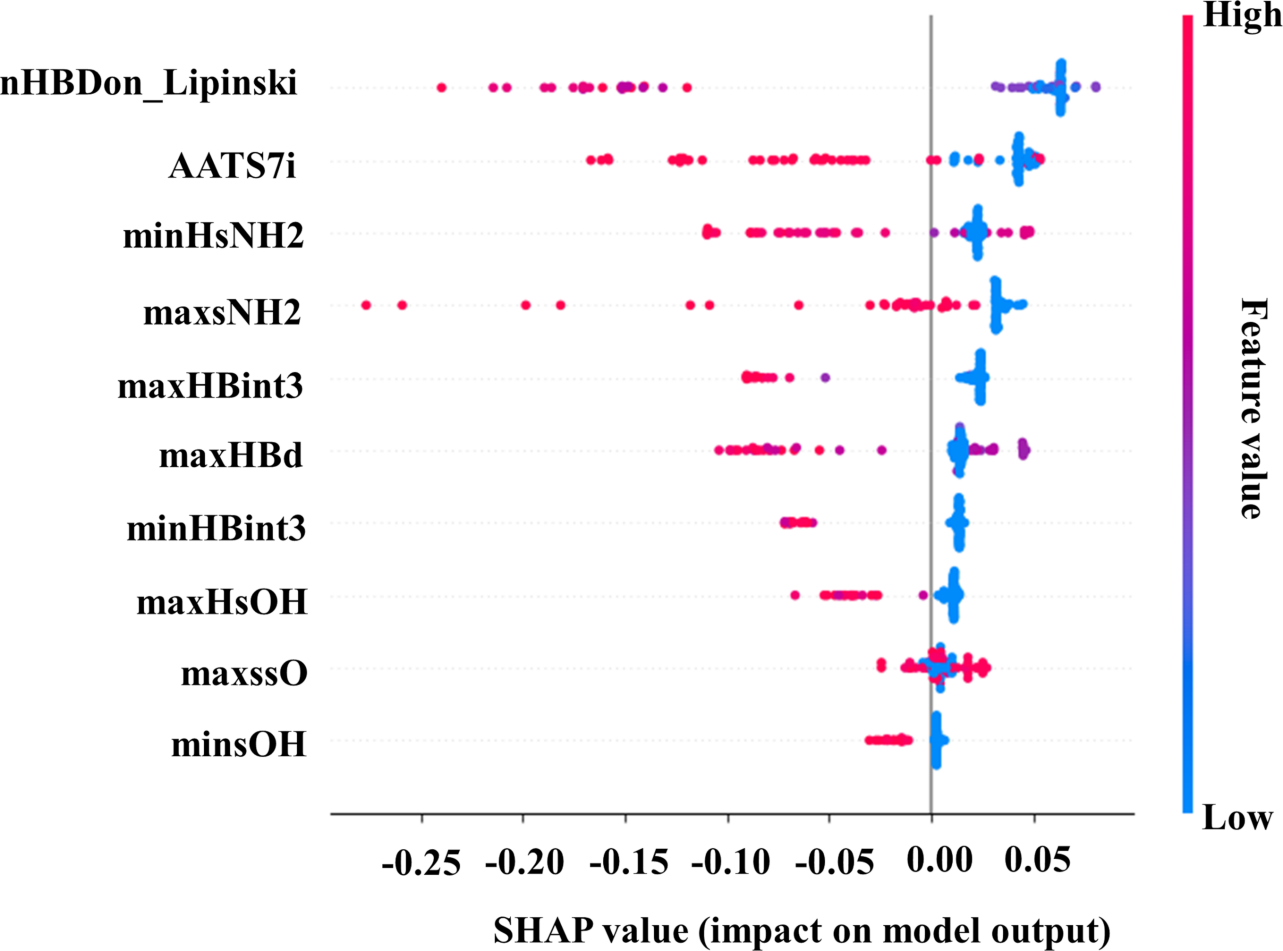
SHAP summary plot for the ML-based RFC model (training set) in the case of PaCa2 cell line.

The SHAP summary plot for the classification random forest model of cellular uptake data of ENMOs in the PaCa2 cell line is shown in Figure 7. The descriptors nHBDon_Lipinski, AATS7i, minHsNH2, maxsNH2, maxHBint3, maxHBd, minHBint3, maxHsOH, maxssO, and minsOH are mentioned in descending order of importance. The details of the descriptors along with their definitions are given in Supporting Information File 1, Table S2.

nHBDon_Lipinski was identified as the most highly contributing feature in the developed model for the PaCa2 cell line. The descriptor nHBDon_Lipinski is associated with Lipinski’s “rule of five” where “nHBDon” stands for the number of hydrogen bond donors present in a molecule [50]. Hydrogen bonds play an important role in interactions between molecules in various biological processes. However, for cellular uptake in the PaCa2 cell line, the contribution of hydrogen bonds has a negative impact as shown in Figure 7. A higher value of nHBDon_Lipinski leads to lower chances of cellular uptake of ENMOs (e.g., **92**, **97**, and **99**). The second significant descriptor according to SHAP analysis (Figure 7) is AATS7i. The descriptor AATS7i is an averaged Moreau–Broto autocorrelation of lag 7 weighted by ionization potential. This descriptor adds the ionization potential with the Moreau–Broto autocorrelation to measure the structural and electronic properties of surface modifiers [51] and has a negative impact on the cellular uptake of ENMOs. For example, in the case of surface modifiers **11**, **24**, **59**, and **97**, higher values of the AATS7i descriptor result in a lower cellular uptake of ENMOs in the PaCa2 cell line. Conversely, surface modifiers **2**, **4**, **17**, and **20** show higher cellular uptake of ENMOs in the PaCa2 cell line while having low values of the AATS7i descriptor. The next descriptor according to SHAP analysis is minHsNH2, which refers to the minimum atom-type E-state indices for the amino (–NH_2_) hydrogens in a molecule [52]. It is observed that the surface modifiers **87**, **88**, **94**, and **98**, which have a higher value of the minHsNH2 descriptor, are not suitable as structural modifiers of ENMOs for the higher cellular uptake in the PaCa2 cell line. Surface modifiers **8**, **17**, and **20** cause higher cellular uptake of the ENMOs in the PaCa2 cell line, and they have a value of zero for the descriptor minHsNH2. Thus, based on the outcomes of the previous Bayesian classification model (UI_p_ 10, UI_p_ 13, and UI_p_ 14 fingerprints in Figure 3) and the current machine learning analyses (Figure 7), it can be concluded that the presence of an amino group in the structure of surface modifiers of ENMOs is not conducive to higher cellular uptake in the PaCa2 cell line. The fourth negatively contributing descriptor in the model output was maxsNH2. In simple terms, the maxsNH2 value indicates the maximum electronic state value of a single-bonded NH_2_ group [53]. It is observed that the structures of surface modifiers **74**, **77**, and **93** are not suitable for higher cellular uptake of ENMOs in the PaCa2 cell line because of the increased maxsNH2 values. Conversely, the values of maxsNH2 in compounds **1**, **2**, and **4** are zero, and these surface modifiers lead to higher cellular uptake in the PaCa2 cell line. This is also suggested by our previous Bayesian classification model (UI_p_ 2 and UI_p_ 3 fingerprints in Figure 3). The fifth negatively contributing descriptor in the model output is maxHBint3 [54]. The increased maxHBint3 values of surface modifiers **87**, **88**, and **94** indicate that the latter are not suitable for higher cellular uptake of ENMOs in the PaCa2 cell line. The descriptor maxHBd signifies the maximum E-states for (strong) hydrogen bond donors [55] and contributes negatively to model output (Figure 7). The next negatively contributing descriptor is minHBint3. Basically, minHBint3 means the minimum E-state descriptors of strength for prospective hydrogen bonds separated by three edges [56]. The negative impact of this descriptor is reinforced by examining compounds **2**, **4**, **8**, and **14**, where the zero value of the minHBint3 descriptor correlates with higher cellular uptake of ENMOs in the PaCa2 cell line. The negatively contributing descriptor maxHsOH refers to the maximum atom-type E-state indices for the hydroxy (–OH) hydrogen in a molecule [57]. The negative contribution is supported by the observation of our previous Bayesian classification model (UI_p_ 12, UI_p_ 15, and UI_p_ 16 fingerprints in Figure 3). The surface modifiers **1**, **2**, **8**, and **17** are characterized by a zero value of the maxHsOH descriptor and are very much suitable for achieving higher cellular uptake of ENMOs in the PaCa2 cell line. The descriptor maxssO denotes the maximum electronic states of the ether-type oxygen (–O–) present in the structure of a compound [58]. It has been observed that the surface modifiers **23**, **29**, and **49**, which have a higher value of the maxssO descriptor, are suitable for the higher cellular uptake of ENMOs in the PaCa2 cell line. In our previous Bayesian classification analysis, we identified similar favorable fingerprints (UP_p_ 11, UP_p_ 12, UP_p_ 13, and UP_p_ 14 fingerprints in Figure 3) for the cellular uptake of ENMOs in the PaCa2 cell line. The descriptor minsOH [59] makes a negative contribution to the final ML model. The descriptor minsOH stands for minimum electronic state value for the single bonded hydroxy group (–OH) present in a structure. It has been observed that the surface modifiers **30**, **78**, and **79**, which have a higher value of the minsOH descriptor, are not suitable for the cellular uptake of ENMOs in the PaCa2 cell line.

#### HUVEC cell line

**Figure 8:**
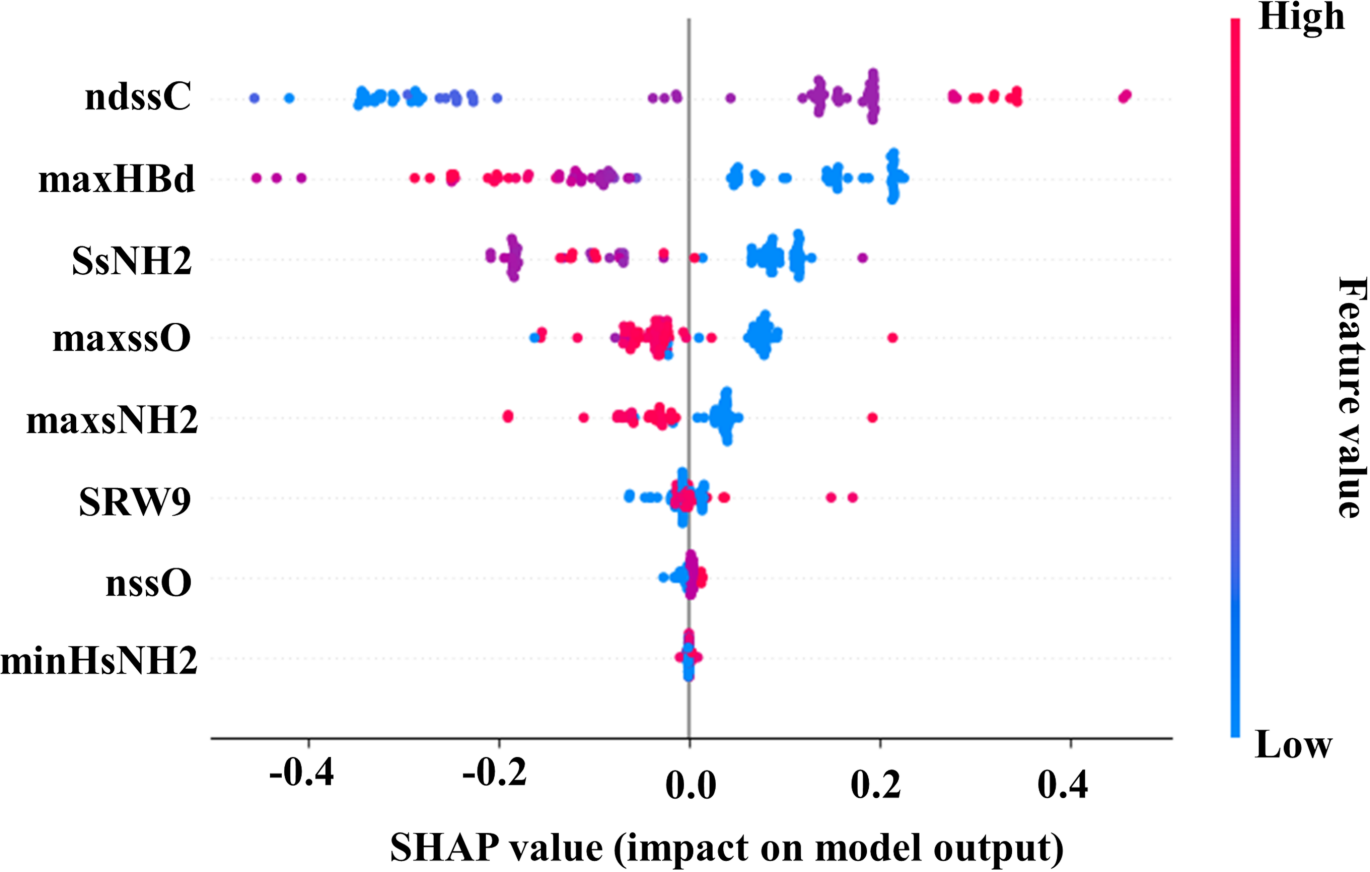
SHAP summary plot for the ML-based SVC model (training set) for the HUVEC cell line.

SHAP analysis on the training dataset of the ML-based support vector classification model for the cellular uptake in HUVEC cell line was performed for the identification of descriptors (Supporting Information File 1, Table S3) to the final model output (Figure 8). Figure 8 shows the important descriptors ndssC, maxHBd, SsNH2, maxssO, maxsNH2, SRW9, nssO, and minHsNH2 in descending order.

Descriptor ndssC is recognized as the most contributing descriptor in the developed model and it denotes the total number of double bonded carbons present in the structure [60]. The positive contribution of the descriptor is confirmed by the presence of maximum double-bonded carbons in the structures (e.g., **39**, **43**, and **46**), which actively contribute to a higher cellular uptake of ENMOs in the case of the HUVEC cell line. From the earlier Bayesian analysis, it was also identified that certain favorable fingerprints (UP_h_ 2 and UP_h_ 14 fingerprints in Figure 4) include a double-bonded carbon in the structure for better cellular uptake.

The descriptor maxHBd indicates the maximum E-States for (strong) hydrogen bond donors [55] and contributes negatively to model output (Figure 8). For example, surface modifiers **88**, **94**, **98**, and **100** are not appropriate for increasing the cellular uptake of ENMOs in the HUVEC cell line, indicated by their high maxHBd values. The third most contributing descriptor was SsNH2. In simple terms, the SsNH2 value indicates the summation value of the electronic state of a single-bonded NH_2_ group present in a compound [61]. Higher values of SsNH2 have a negative impact on the cellular uptake of ENMOs in the HUVEC cell line (e.g., **71**, **76**, **80**, and **92**). The Bayesian classification model also revealed that fingerprints UI_h_ 7, UI_h_ 8, and UI_h_ 9 in Figure 4, containing an NH_2_ group, are unsuitable as structural modifiers of ENMOs for higher uptake in the HUVEC cell line.

The next descriptor that has been identified for its negative contribution is maxssO*.* The descriptor maxssO denotes the maximum electronic states of the ether-type oxygen (–O–) present in the structures of a compound [58]. It has been observed in most of the cases that the surface modifiers **15**, **18**, **27**, and **37**, which have a higher value of the maxssO descriptor*,* are not suitable for the higher cellular uptake of ENMOs in the HUVEC cell line. The fifth negatively contributing descriptor in the model output was maxsNH2. The maxsNH2 value indicates the maximum electronic state value of a single-bonded NH_2_ group [53]. Higher values of maxsNH2 lead to a lower cellular uptake of ENMOs in the HUVEC cell line (e.g., **1**, **2**, **14**, and **104**). The aforementioned observation was previously noted in the Bayesian classification analysis, where certain unfavorable fingerprints (UI_h_ 7, UI_h_ 8, and UI_h_ 9 fingerprints in Figure 4) containing an NH_2_ group in their structure were identified. The other descriptors like SRW9 [62], nssO [63], and minHsNH2 have lower contribution in the model for the cellular uptake of ENMOs in the HUVEC cell line.

#### U937 cell line

**Figure 9:**
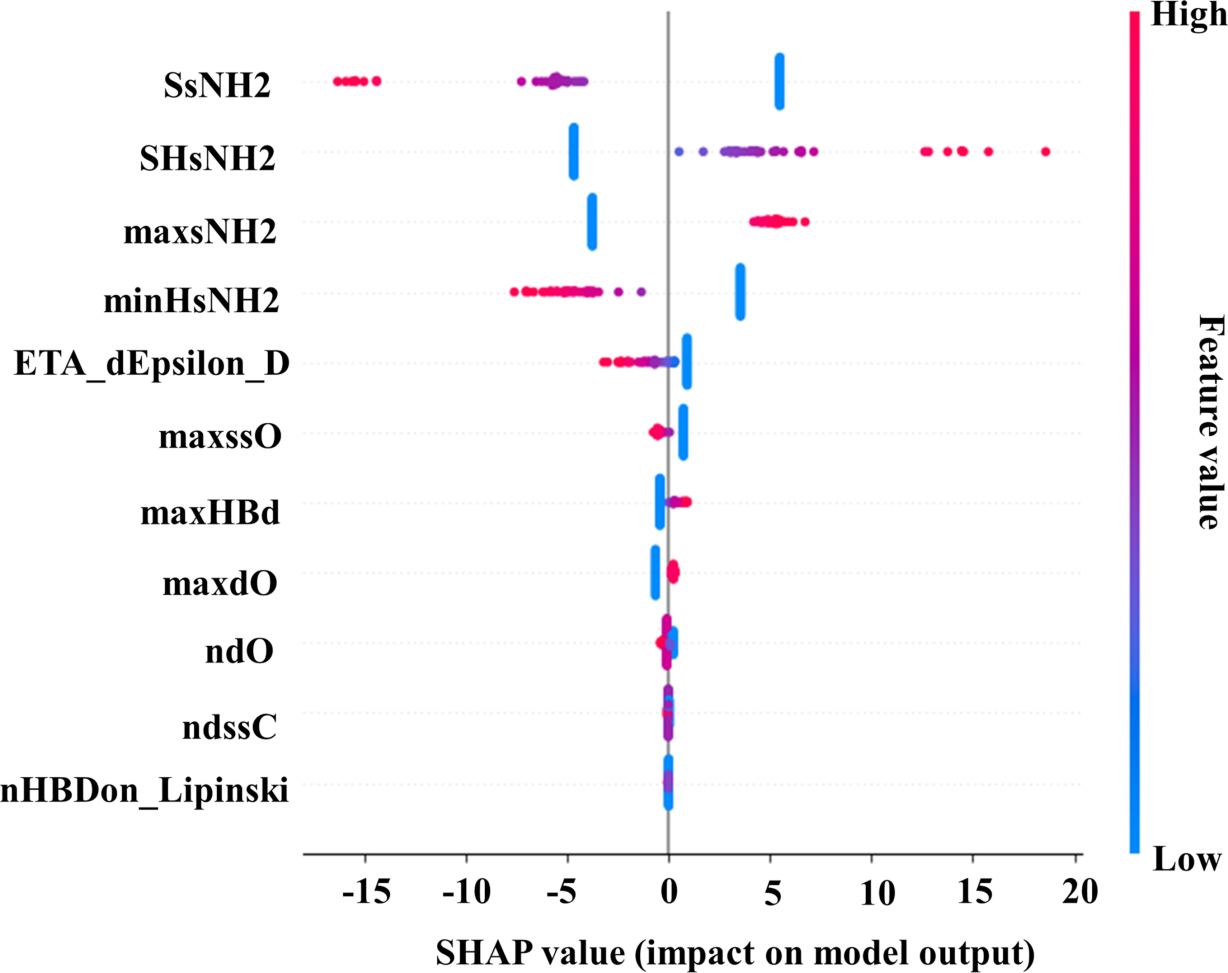
SHAP summary plot for the ML-based LDA model (training set) in the case of the U937 cell line.

We performed SHAP analysis regarding the U937 cell line, and the plot is shown in Figure 9. The details of descriptors definitions are explained in Supporting Information File 1, Table S4.

The descriptor SsNH2 is recognized as the most contributing feature in the developed model. In simple terms, the SsNH2 value indicates the summation value of the electronic state of a single-bonded NH_2_ group present in a compound [61]. Higher values of SsNH2 have a negative impact on the cellular uptake of ENMOs in the U937 cell line (e.g., **69**, **71**, and **80**). The next, positively contributing, descriptor is SHsNH2, calculated as the sum of the atom-type E-state indices for all –NH_2_ hydrogens in a molecule [64]. The variable maxsNH2 makes a significant positive contribution to the model (Figure 9). The descriptor maxsNH2 refers to the maximum electronic state value for the single-bonded NH_2_ group present in a structure [53]. It is noticed in the cases of surface modifiers **77** and **86** that these structures are suitable for higher cellular uptake of ENMOs in the U937 cell line*.* The descriptor minHsNH2 exhibited a negative contribution to the final model output. The minHsNH2 descriptor refers to the minimum atom-type E-state indices for all of the amino (–NH_2_) hydrogens in a molecule [52]. It is observed that the surface modifiers **94** and **98**, which have a higher value of the minHsNH2 descriptor, are not suitable as structural modifiers of ENMOs for the higher cellular uptake in the U937 cell line. The descriptor ETA_dEpsilon_D [65] signifies that surface modifiers containing a higher number of strongly electronegative atoms (such as N, O, and F) or hydrogen bond donor atoms will cause a lower uptake of ENMOs in the U937 cell line (e.g., **6**, **9**, and **15**). Other descriptors including maxssO, maxHBd, maxdO, ndO, ndssC, and nHBDon_Lipinski contribute less to the cellular uptake of ENMOs in the U937 cell line.

## Conclusion

**Figure 10:**
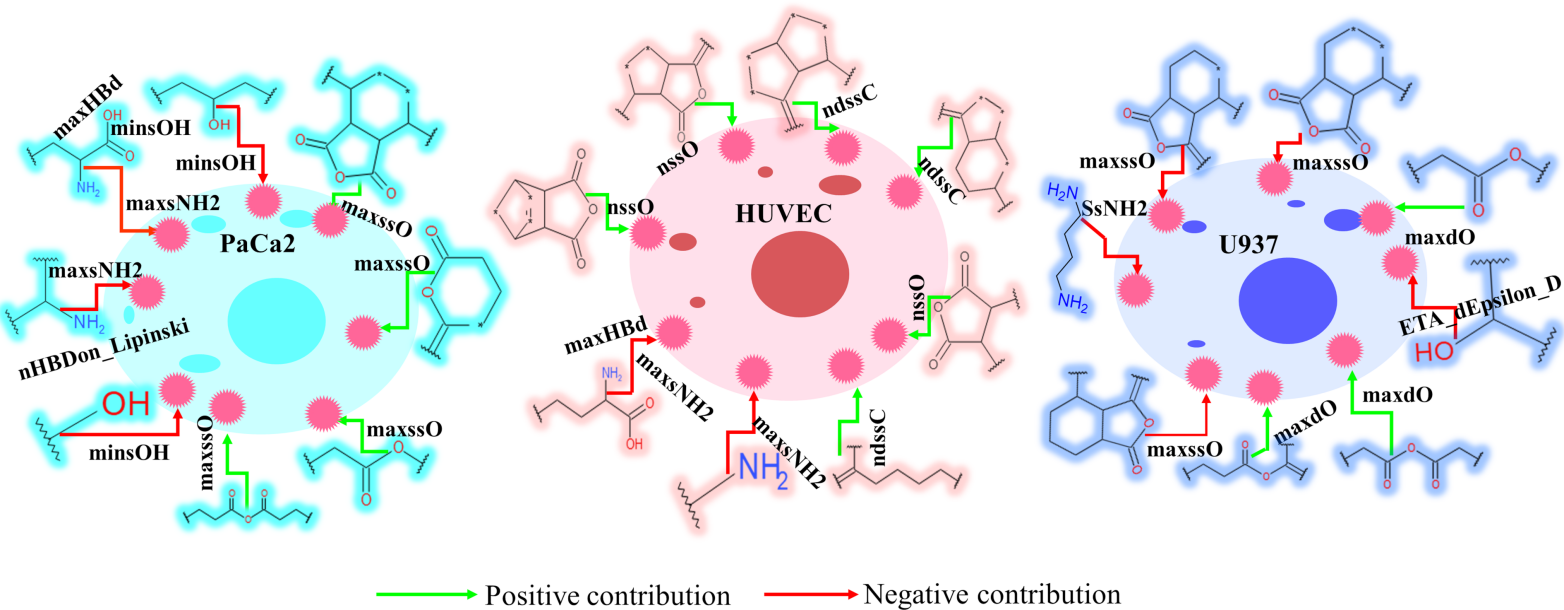
Summary of structural features of surface modifiers of ENMOs for the uptake in the PaCa2, HUVEC, and U937 cell line models.

Identifying the surface modifiers of engineered nanostructured metal oxides (ENMOs) that enhance affinity for certain cell types while reducing uptake by non-target cells could significantly improve the efficacy of targeted therapies and minimize off-target effects. In this study, classification-based machine learning models have been created separately using cellular uptake data from 109 surface modifiers of ENMOs in three cell lines, namely, PaCa2, HUVEC, and U937, for the identification of distinctive fingerprints/descriptors controlling the cellular uptake in the specific cell line. Significant uptake-promoting and uptake-impairing fingerprints were identified for different cell lines based on Bayesian classification studies. The best machine learning (ML) model for the PaCa2 cell line was the random forest (RF), which achieved fivefold cross-validated ROC values of 0.939 for the training set and 0.818 for the test set, indicating acceptable internal and external validation results. Similarly, the best-performing ML model for the HUVEC cell line was support vector classifier (SVC), which demonstrated fivefold cross-validated ROC values of 0.969 for the training set and 0.870 for the test set, indicating successful internal and external validation. Finally, the top ML model for the U937 cell line, linear discriminant analysis (LDA), yielded fivefold cross-validated ROC values of 0.735 for the training set and 0.630 for the test set. The findings revealed distinctive structural fingerprints associated with the cellular uptake of nanoparticles in each cell line (Figure 10). For example, the presence of a hydroxy group in the structures of the surface modifiers leads to a decrease in the cellular uptake of ENMOs in the PaCa2 cell line only. Furthermore, the study also identifies some common structural fingerprints among surface modifiers (Supporting Information File 1, Figures S7–S8) observed in uptake across multiple cell lines. It is observed from SHAP analysis that there are three major descriptors (maxsNH2, maxHBd, and maxssO) identified as common in the three best ML models developed for the three different cell lines. Having one or more aliphatic primary amino groups (descriptor maxsNH2) in the surface modifiers leads to reduced cellular uptake of ENMOs in both PaCa2 and HUVEC cell lines. Neither does a higher number of hydrogen bond donating groups (descriptor maxHBd) in the surface modifiers promote greater cellular uptake of ENMOs in these cell lines. Additionally, the study highlights that the presence of ether-type oxygen (descriptor maxssO) in the surface modifier structure may contribute to increased cellular uptake across the three cell lines. The structural fingerprints/descriptors obtained from the current modelling study will be helpful to scientists for the future design of surface modifiers of nanostructured metal oxides. This may facilitate a higher therapeutic response by surface modifier-mediated site-specific targeting to the cell surface receptors of particular cell types. Further availability of sufficient and reliable uptake data of ENMOs in other cell types is also needed for better confirmation of these fingerprints/descriptors in the design of surface modifiers of ENMOs.

## Supporting Information

File 1 Additional figures and tables.

## Data Availability

All data that supports the findings of this study is available in the published article and/or the supporting information to this article.
