# Supplementary material for: Identification of structural features of surface modifiers in engineered nanostructured metal oxides regarding cell uptake through ML-based classification
Source: Beilstein J Nanotechnol. 2024 Jul 22;15:909–24. doi: 10.3762/bjnano.15.75 (PMC11285082; doi:10.3762/bjnano.15.75)
Supplement: File 1 — Additional figures and tables. [file Beilstein_J_Nanotechnol-15-909-s001.pdf]

BEILSTEIN JOURNAL OF NANOTECHNOLOGY

Supporting Information

for

Identification of structural features of surface modifiers in engineered nanostructured metal oxides regarding cell uptake through ML-based classification

Indrasis Dasgupta, Totan Das, Biplab Das and Shovanlal Gayen

Beilstein J. Nanotechnol. **2024,** *15,* 909–924. doi:10.3762/bjnano.15.75

Additional figures and tables

License and Terms: This is a supporting information file under the terms of the Creative Commons Attribution License (https://creativecommons.org/licenses/by/4.0). Please note that the reuse, redistribution and reproduction in particular requires that the author(s) and source are credited and that individual graphics may be subject to special legal provisions.
The license is subject to the *Beilstein Journal of Nanotechnology* terms and conditions: (https://www.beilstein-journals.org/bjnano/terms)

Table S1: Dataset of 109 surface modifiers of magnetofluorescent ENMOs (monocrystalline magnetic nanoparticle having overall size of 38 nm)

		PaCa2[a]		HUVEC[b]		U937[c]	
No.	Smiles notation of the surface modifiers	log10 [ENMO]/cell	Binary	log10 [ENMO]/cell	Binary	log10 [ENMO]/cell	Binary
1	C(C(=O)OC(=O)C(F)(F)F)(F)(F)F	4.17	1	4.40	1	3.04	1
2	FC(F)(Cl)C(=O)OC(=O)C(F)(F)Cl	3.95	1	4.08	1	3.01	1
3*	C(C(F)(F)F)(C(=O)OC(=O)C(C(F)(F)F)(F)F)(F)F	4.08	1	4.53	1	2.96	0
4	CC1(C)CC(=O)OC1=O	4.11	1	3.94	1	2.96	0
5*	O1C(=O)C=CC1=O	3.98	1	4.00	1	3.02	1
6	O1C(=O)C(=CC1=O)C	3.58	0	4.04	1	3.11	1
7	O1C(=O)C(=C(C1=O)C)C	3.48	0	4.63	1	2.98	1
8	C(CCCC)C(=O)OC(=O)CCCCC	3.65	1	3.91	1	3.01	1
9	C1(=O)OC(=O)[C@@H](C1)C	3.64	0	3.17	0	3.00	1
10*	C(=O)([C@@H]1C[C@H]2C(=O)OC(=O)[C@@H]2CC1)[C@H]1C[C@H]2C(=O)OC(=O)[C@H]2CC1	3.51	0	4.67	1	3.00	1
11	C1(=O)OC(=O)[C@@H]2C[C@H](CC[C@@H]12)[N+](O)[O-]	3.27	0	4.57	1	3.02	1
12	Brc1ccc2C(=O)OC(=O)c3cccc1c23	3.63	0	4.03	1	3.07	1
13*	O=C1OC(=O)c2ccc3C(=O)OC(=O)c4ccc1c2c34	3.67	1	3.21	0	2.95	0
14	C1(=O)OC(=O)[C@H]2[C@H]([C@@H]([C@H]([C@@H]([C@H]12)F)F)F)F	3.83	1	4.39	1	3.00	1
15	O=C1OC(=O)c2cc(cc3cccc1c23)N(O)O	4.11	1	3.22	0	3.08	1
16	C1(=O)OC(=O)[C@@H]2[C@@H](CCC[C@H]12)O	3.97	1	3.25	0	2.96	0
17	O=C1OC(=O)[C@@H]2[C@H]3CC[C@H](C=C3)[C@H]12	3.9	1	4.54	1	3.01	1
18	[C@@H]12NC(=O)OC(=O)[C@@H]1C[C@@H](CC2)Cl	4.18	1	2.88	0	3.07	1
19*	[C@@H]12S(=O)(=O)OC(=O)[C@@H]1CCCC2	3.88	1	4.40	1	2.98	1
20	O1C(=O)C(=C(C1=O)Cl)Cl	3.84	1	4.47	1	3.01	1
21	CC(=O)S[C@@H]1C(=O)OC(=O)C1	3.59	0	3.75	1	3.02	1
22	C1(=O)OC(=O)[C@@H]2C[C@H]([C@@H](C[C@@H]12)Cl)Cl	4.12	1	4.48	1	3.00	1
23	O=C1OC(=O)[C@H]2[C@H]3O[C@H](C=C3)[C@@H]12	3.82	1	4.11	1	2.98	1
24	O=C1OC(=O)[C@H]2[C@@H]3C=C[C@H]([C@@H]12)[C@@H]1[C@H]3C(=O)OC1=O	3.63	0	4.35	1	3.00	1
25	C1(=O)OC(=O)[C@H]2CC=CC[C@@H]12	3.89	1	3.76	1	3.03	1
26*	O1C(=O)[C@H]2[C@H](CCCC2)[C@@H]2[C@H](C1=O)CCCC2	3.77	1	4.70	1	2.99	1
27	O=C1OC(=O)c2ccc(c3cccc1c23)N(O)O	3.93	1	3.34	0	3.08	1
28	O=C1OC(=O)[C@H]2[C@@H]1[C@H]1[C@@H]2C(=O)OC1=O	3.77	1	3.82	1	3.07	1
29	CCCCCCCCCCCC(=O)OC(=O)CCCCCCCCCCC	3.82	1	4.52	1	3.02	1

30	C1(=O)OC(=O)[C@@H]2C[C@H](CC[C@H]12)C(=O)O	3.55	0	4.21	1	3.05	1
31	C1(=O)OC(=O)[C@@H]2C[C@H](CC[C@H]12)C	3.98	1	4.30	1	3.02	1
32	C1(=O)OC(=O)[C@H]2[C@@H](CCC[C@@H]12)[N+](O)[O-]	3.5	0	4.45	1	2.96	0
33	C1(=O)OC(=O)C[C@H]2CCCC[C@@H]12	3.78	1	4.18	1	2.96	0
34	O1C(=O)CCCC1=O	4.07	1	3.41	0	3.00	1
35	C(CN1CC(=O)OC(=O)C1)N1CC(=O)OC(=O)C1	3.93	1	4.00	1	2.94	0
36	[C@H]12NC(=O)OC(=O)[C@@H]1CCCC2	4.44	1	3.59	0	3.12	1
37	[C@@H]12N(C(=O)OC(=O)[C@@H]1CCCC2)C	3.36	0	2.15	0	2.85	0
38	O1C(=O)CC(CC1=O)C	3.91	1	3.06	0	2.98	1
39	C1(=O)OC(=O)C2=C1CCCC2	3.73	1	4.57	1	3.00	1
40*	CC(=O)O[C@@H]1[C@@H](OC(=O)C)C(=O)OC1=O	3.91	1	4.58	1	3.04	1
41*	C1(=O)OC(=O)[C@H]2[C@@H]([C@H]([C@@H]([C@H]([C@H]12)Br)Br)Br)Br	3.8	1	4.57	1	3.07	1
42	O=C1OC(=O)[C@H]2CCCC[C@H]12	3.93	1	3.29	0	2.99	1
43	O1C(=O)C2=C(C1=O)CCC2	3.69	1	4.31	1	3.02	1
44*	C(C(=O)OC(=O)CI)I	3.42	0	4.24	1	2.80	0
45	C(C(=O)OC(=O)CCl)Cl	3.63	0	4.56	1	2.99	1
46	ClC1=C(Cl)[C@@]2(Cl)[C@H]3[C@H](C(=O)OC3=O)[C@]1(Cl)C2(Cl)Cl	3.47	0	4.60	1	2.96	0
47*	CCCCCCCCCCCCCCCC(=O)OC(=O)CCCCCCCCCCCCCCC	3.55	0	4.68	1	2.97	0
48	Nc1ccc2C(=O)OC(=O)c3cccc1c23	3.64	0	4.64	1	2.97	0
49	C(CCCCCCCC)C(=O)OC(=O)CCCCCCCCC	4.03	1	4.73	1	3.12	1
50	C1CCCC21CC(=O)OC(=O)C2	4.06	1	3.71	0	3.09	1
51	O=C1OC(=O)[C@@H]2[C@H]3C=C[C@H](C3)[C@H]12	3.94	1	4.37	1	3.01	1
52*	O=C1OC(=O)c2cccc3cccc1c23	3.96	1	4.09	1	3.04	1
53	O1C(=O)[C@@H](CCC1=O)C1CCCCC1	4.02	1	3.90	1	3.02	1
54	C1(=O)OC(=O)[C@@H]2[C@H]([C@@H]([C@@H]([C@H]([C@H]12)Cl)Cl)Cl)Cl	3.83	1	4.76	1	3.04	1
55	C1(=O)OC(=O)[C@H]2[C@@H](CC[C@@H]([C@@H]12)Cl)Cl	3.9	1	4.70	1	2.95	0
56	O1C(=O)C(CCC1=O)(C)C	3.94	1	3.28	0	2.97	0
57	C(CCCC)N	3.78	1	3.31	0	2.95	0
58	C[C@H](CC(C)C)N	3.85	1	4.14	1	3.03	1
59	[C@H]1([C@H]([C@@H]([C@H](C[C@H]1CO)O)N)O)O	3.36	0	3.28	0	2.89	0
60*	C(CCCCC)N	3.75	1	3.34	0	3.00	1
61	CC(C)(N)C	3.86	1	3.34	0	2.97	0
62	C(C(C)C)N	3.72	1	3.08	0	2.92	0
63	[C@H](C(C)C)(N)C	3.75	1	2.97	0	2.88	0

64	C(CC(C)C)N	3.83	1	3.19	0	2.94	0
65	CCC(CC)N	3.81	1	3.32	0	3.03	1
66	CC(CC)(N)C	4.07	1	3.91	1	3.02	1
67	C(CN)N	3.46	0	3.08	0	2.91	0
68	C(CCCCCCCCCCCCCC)N	4.06	1	3.49	0	3.26	1
69	C(CCN)N	3.49	0	2.96	0	2.73	0
70	C(CCCN)N	3.48	0	3.09	0	2.93	0
71	C(CCCCCN)N	3.62	0	3.05	0	2.93	0
72	C([C@H](CCCC)CC)N	3.95	1	3.30	0	3.28	1
73	C(CCCCCCCCCCCCCCC)N	3.97	1	3.27	0	3.07	1
74	C[C@H](CCCCC)N	3.63	0	2.97	0	2.89	0
75*	C(CCCCCCCCCCCCC)N	4.27	1	3.55	0	3.44	1
76	C(CN)NCCN	3.77	1	3.41	0	2.73	0
77	NC[C@@]12C[C@@H]3C[C@@H](C[C@@H](C3)C1)C2	2.84	0	3.38	0	3.09	1
78	[C@@H]1([C@H](C[C@H](CC1)CCN)O)O	2.53	0	2.96	0	2.92	0
79	[C@@H]1(CC[C@H](CC1)CCN)O	2.77	0	3.32	0	2.77	0
80	C(CCCNCCCN)NCCCN	2.41	0	3.35	0	2.57	0
81*	C(CCNCCN)NCCN	2.23	0	3.37	0	2.75	0
82*	C(CNCCNCCNCCNCCN)N	2.54	0	3.21	0	2.73	0
83	N[C@@]12C[C@@H]3C[C@@H](C[C@H]1C3)C2	3.12	0	3.25	0	2.80	0
84*	N[C@@H]1[C@H]2C[C@@H]3C[C@H](C2)C[C@H]1C3	3.18	0	3.31	0	2.93	0
85*	C(C(=O)O)N	2.57	0	3.44	0	2.73	0
86	[C@@H](CC1CCCCC1)(C(=O)OC)N	3.39	0	3.22	0	3.40	1
87	[C@@H](CO)(C(=O)O)N	3.36	0	3.07	0	3.06	1
88	[C@@H]([C@@H](C)O)(C(=O)O)N	3.21	0	2.85	0	3.00	1
89*	[C@@H](C[C@@H]1CN[C@H]2[C@@H]1CCCC2)(C(=O)O)N	3.19	0	3.11	0	3.25	1
90	[C@@H](C[C@H]1CC[C@@H](CC1)O)(C(=O)O)N	3.07	0	2.93	0	2.95	0
91	[C@@H](C(C)C)(C(=O)O)N	3.27	0	3.02	0	3.08	1
92	NCCCC[C@H](N)C(=O)O	3.25	0	3.58	0	2.69	0
93	[C@H](C(=O)O)([C@@H]1CC[C@@H](CC1)Cl)N	3.06	0	3.74	0	2.96	0
94	C[C@H](N)C(=O)O	2.9	0	3.30	0	2.81	0
95*	[C@@H](CCCNC(=N)N)(C(=O)O)N	3.15	0	3.37	0	2.93	0
96*	[C@@H](CC(=O)O)(C(=O)O)N	3.29	0	3.77	1	3.03	1
97	[C@@H](CCC(=O)N)(C(=O)O)N	3.32	0	3.66	0	2.99	1
98	N[C@@H](CCC(=O)O)C(=O)O	3.4	0	3.12	0	2.83	0
99	[C@@H](C[C@@H]1NCNC1)(C(=O)O)N	3.38	0	3.01	0	3.02	1
100	[C@@H](CCSC)(C(=O)O)N	3.23	0	3.12	0	2.97	0
101	[C@@H](CC1CCCCC1)(C(=O)O)N	3.29	0	3.10	0	3.12	1
102	O=C1CCC(=O)O1	4.24	1	3.76	1	3.03	1

103	CC(=O)OC(=O)C	4.05	1	3.99	1	3.01	1
104	C=C1CC(=O)OC1=O	4.04	1	4.43	1	2.92	0
105	O1C(=O)COCC1=O	3.99	1	4.23	1	2.98	0
106	C1(=O)OC(=O)[C@@H]2CCCC[C@H]12	3.9	1	4.39	1	2.93	0
107	C(C(=O)O)[C@H]1C(=O)OC(=O)C1	4.03	1	4.64	1	3.02	1
108	C1(=O)OC(=O)[C@H]2[C@@H](CC[C@H]([C@H]12)F)F	3.91	1	4.31	1	2.94	0
109*	C(C(=O)O)N(CCN1CC(=O)OC(=O)C1)CCN1CC(=O)OC(=O)C1	4.1	1	4.26	1	2.98	1

'*' indicates Test Set compounds, [a]log10 [MNP]/cell > 3.646 is assigned as 1 and log10 [MNP]/cell < 3.646 is assigned as 0, [b]log10 [MNP]/cell > 3.758 is assigned as 1 and log10 [MNP]/cell < 3.758 is assigned as 0, = 3.758, [c]log10 [MNP]/cell > 2.985 is assigned as 1 and log10 [MNP]/cell < 2.985 is assigned as 0.

Table S2: Descriptors identified in the study for the cellular uptake of the engineered nano metal oxides (ENMOs) in the PaCa2 (human pancreatic ductal adenocarcinoma cells) cell line.

Name of the descriptors	Description
nHBDon_Lipinski	Number of hydrogen bond donors (using Lipinski's definition: Any OH or NH. Each available hydrogen atom is counted as one hydrogen bond donor)
AATS7i	Average Broto-Moreau autocorrelation - lag 7 / weighted by first ionization potential
minHsNH2	Minimum atom-type H E-State: -NH2
maxsNH2	Maximum atom-type E-State: -NH2
maxHBint3	Maximum E-State descriptors of strength for potential Hydrogen Bonds of path length 3
maxHBd	Maximum E-States for (strong) Hydrogen Bond donors
minHBint3	Minimum E-State descriptors of strength for potential Hydrogen Bonds of path length 3
maxHsOH	Maximum atom-type H E-State: -OH
maxssO	Maximum atom-type E-State: -O-
minsOH	Minimum atom-type E-State: -OH

Table S3: Descriptors identified in the study for the cellular uptake of the engineered nano metal oxides (ENMOs) in the HUVEC (human umbilical vein endothelial cells) cell line.

Name of the descriptors	Description
ndssC	*Count of atom-type E-State: =C<*
maxHBd	*Maximum E-States for (strong) Hydrogen Bond donors*
SsNH2	*Sum of atom-type E-State: -NH2*
maxssO	*Maximum atom-type E-State: -O-*
maxsNH2	*Maximum atom-type E-State: -NH2*
SRW9	*Self-returning walk count of order 9*
nssO	*Count of atom-type E-State: -O-*
minHsNH2	*Minimum atom-type H E-State: -NH2*

Table S4: Descriptors identified in the study for the cellular uptake of the engineered nano metal oxides (ENMOs) in the U937 (human monocyte lymphoma) cell line.

Name of the descriptors	Description
SsNH2	*Sum of atom-type E-State: -NH2*
SHsNH2	*Sum of atom-type H E-State: -NH2*
maxsNH2	*Maximum atom-type E-State: -NH2*
minHsNH2	*Minimum atom-type H E-State: -NH2*
ETA_dEpsilon_D	*A measure of contribution of hydrogen bond donor atoms*
maxssO	*Maximum atom-type E-State: -O-*
maxHBd	*Maximum E-States for (strong) Hydrogen Bond donors*
maxdO	*Maximum atom-type E-State: =O*
ndO	*Count of atom-type E-State: =O*
ndssC	*Count of atom-type E-State: =C<*
nHBDon_Lipinski	*Number of hydrogen bond donors (using Lipinski's definition: Any OH or NH. Each available hydrogen atom is counted as one hydrogen bond donor)*

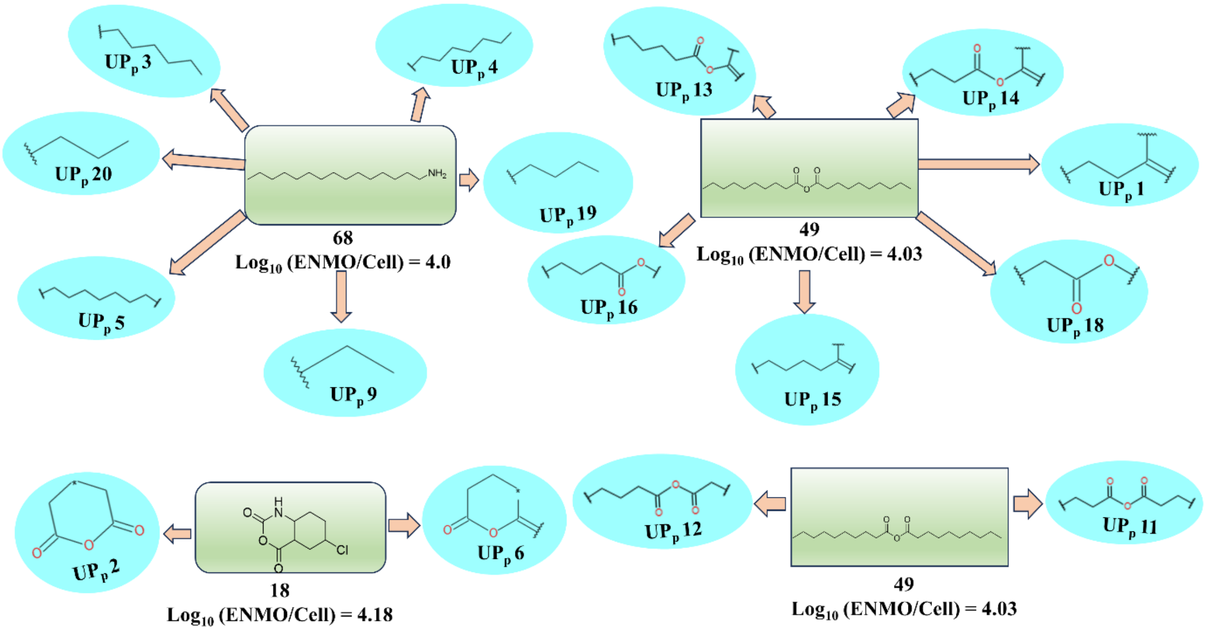

Figure S1: Structures of various surface modifiers (**18**, **49** and **68**) from the dataset containing uptake-promoting fingerprints (PaCa2 cell line).

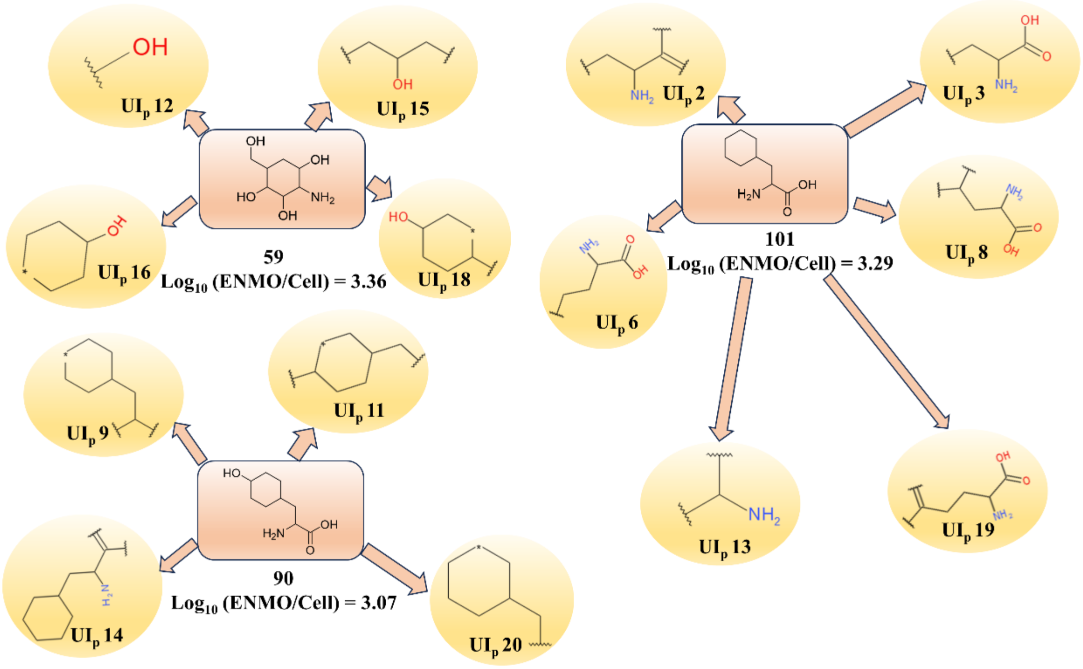

Figure S2: Structures of various surface modifiers (**59**, **90** and **101)** from the dataset containing uptake-impairing fingerprints (PaCa2 cell line).

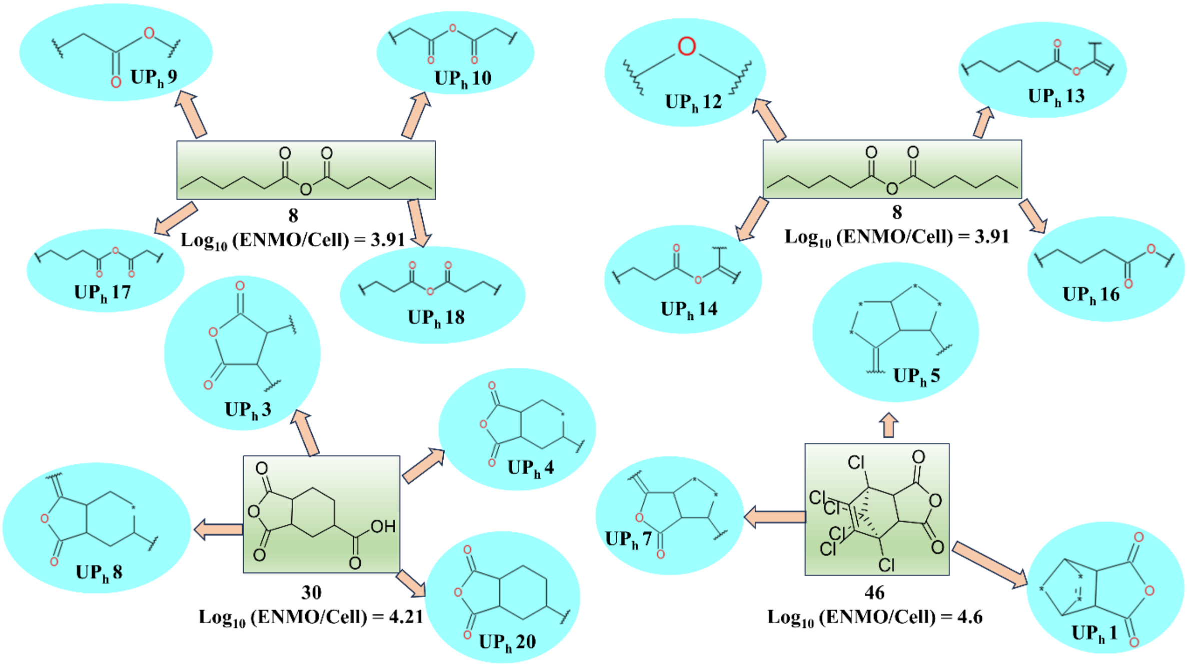

Figure S3: Structures of various surface modifiers (**8**, **30** and **46**) from the dataset containing uptake-promoting fingerprints (HUVEC cell line).

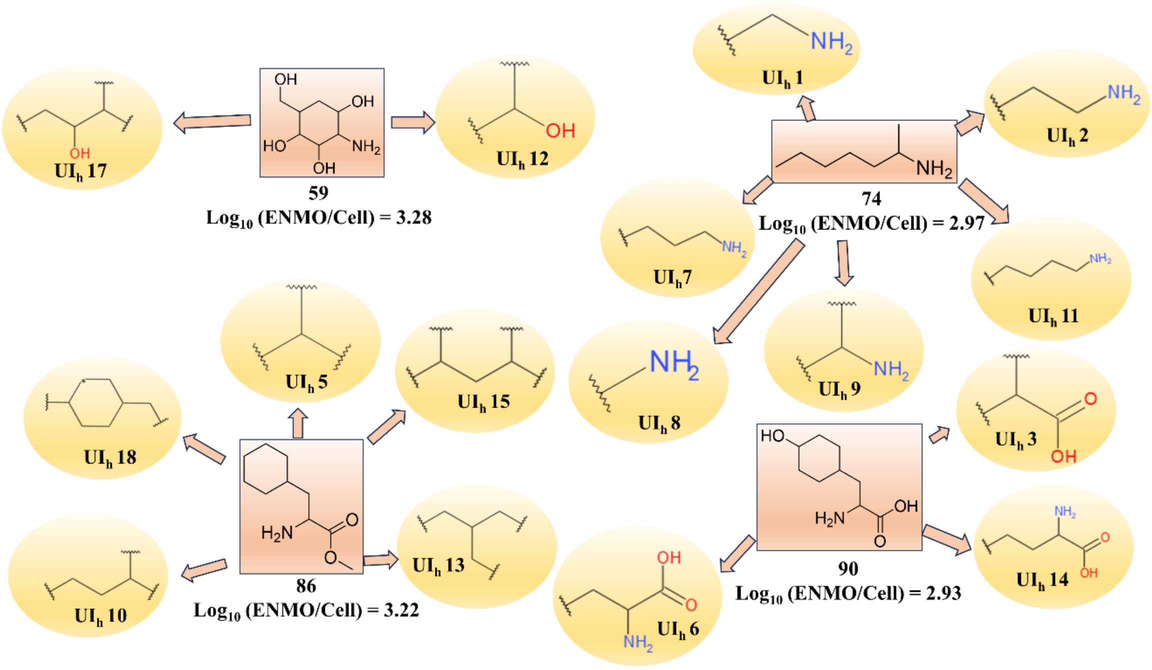

Figure S4: Structures of various surface modifiers (**59**, **74**, **86** and **90**) from the dataset containing uptake-impairing fingerprints (HUVEC cell line).

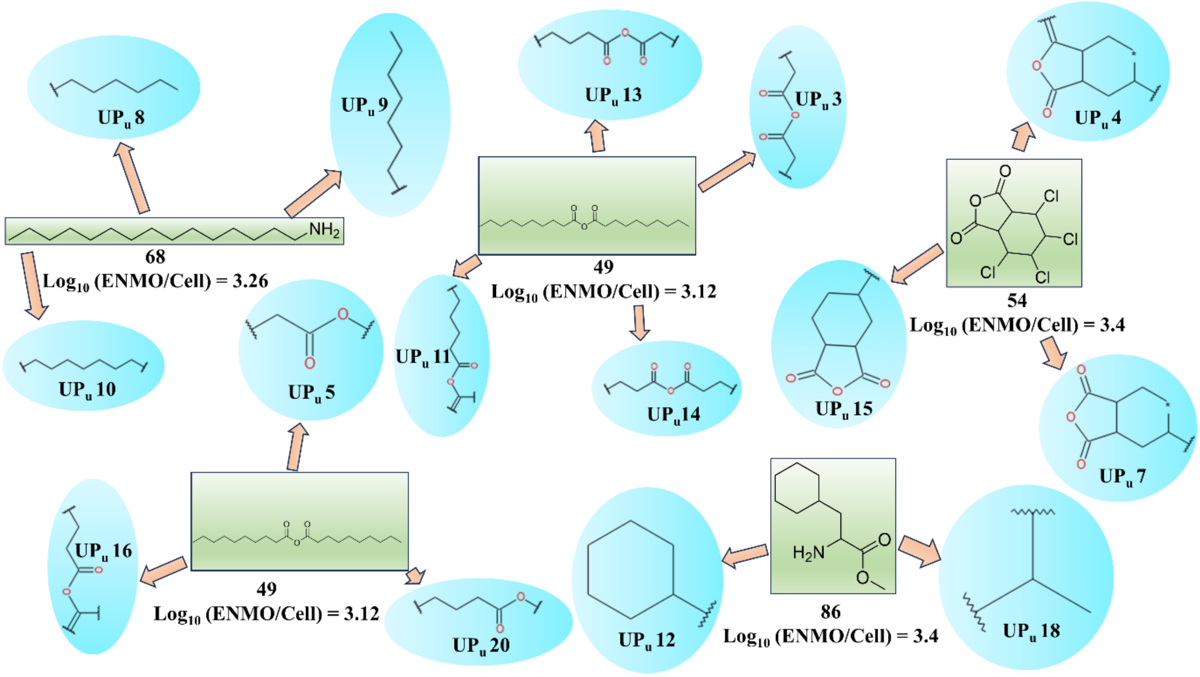

Figure S5: Structures of various surface modifiers (**49**, **54**, **68** and **86**) from the dataset containing uptake-promoting fingerprints (U937 cell line).

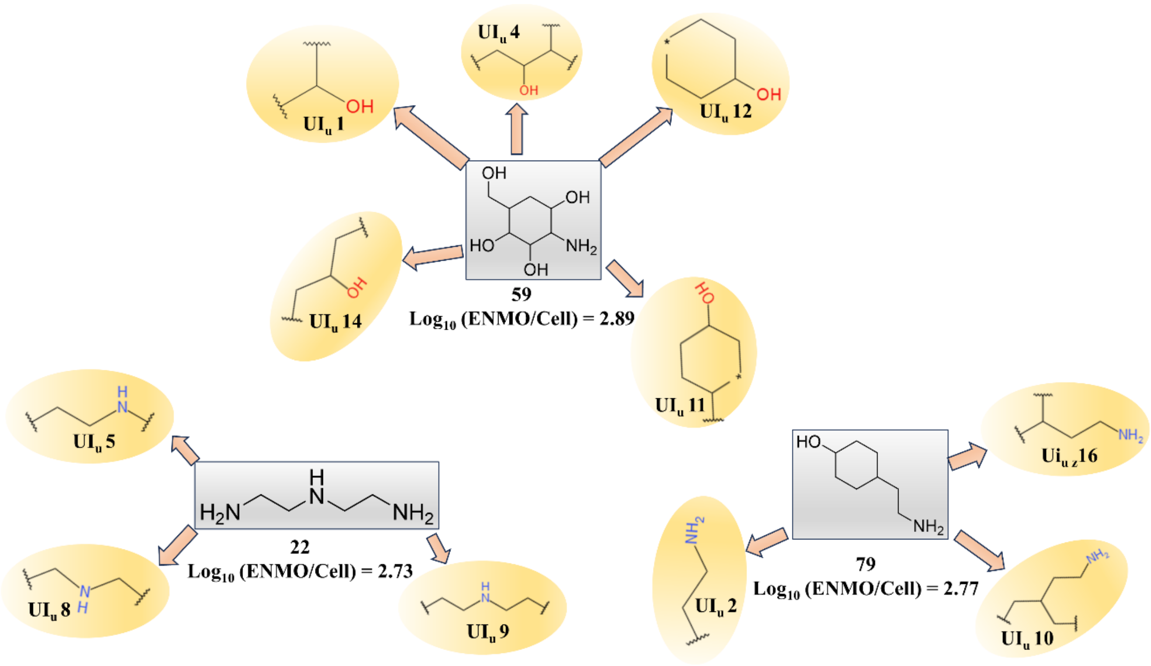

Figure S6: Structures of various surface modifiers (**22**, **59** and **79**) from the dataset containing uptake-impairing fingerprints (U937 cell line).

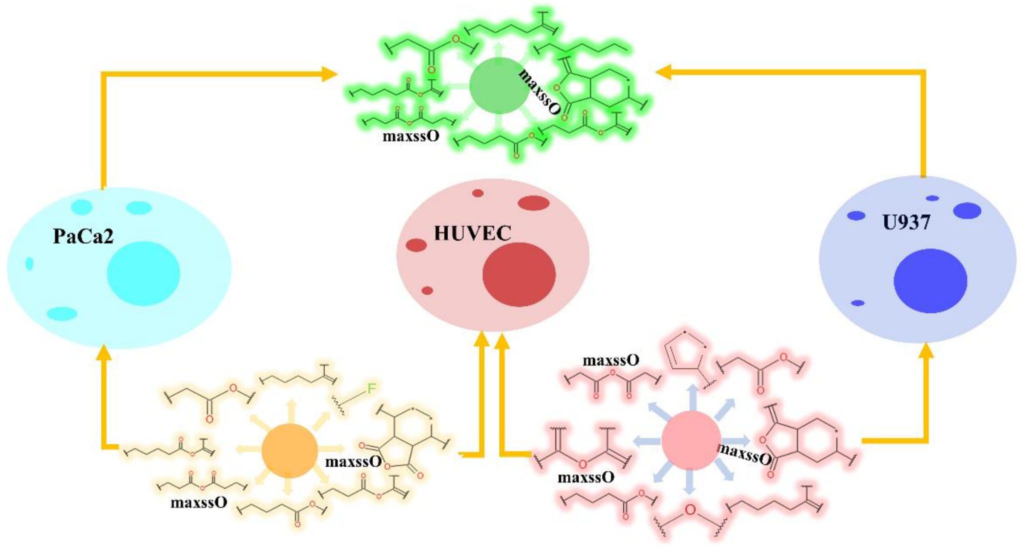

Figure S7: Correlation of common fingerprints obtained from Bayesian and machine learning studies with the three different cell lines (PaCa2, HUVEC, and U937).

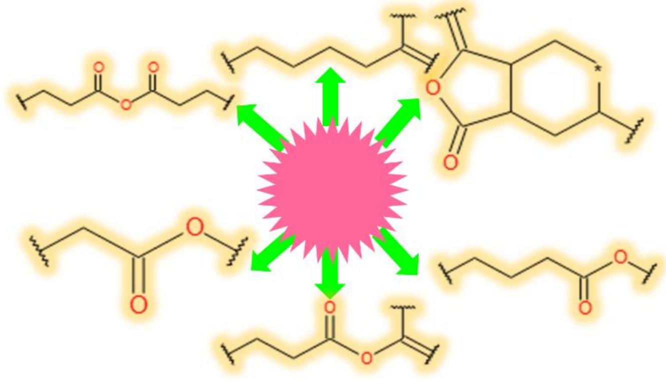

Figure S8: Common fingerprints were obtained from Bayesian classification for the three different cell lines (PaCa2, HUVEC, and U937).
